# SARS-CoV-2 ORF8 and SARS-CoV ORF8ab: Genomic Divergence and Functional Convergence

**DOI:** 10.3390/pathogens9090677

**Published:** 2020-08-20

**Authors:** Sameer Mohammad, Abderrezak Bouchama, Bothina Mohammad Alharbi, Mamoon Rashid, Tanveer Saleem Khatlani, Nusaibah S. Gaber, Shuja Shafi Malik

**Affiliations:** 1 Experimental Medicine Department, King Abdullah International Medical Research Center, King Saud bin Abdulaziz University for Health Sciences, MNGHA, Riyadh 11426, Saudi Arabia; mohammadsa1@ngha.med.sa (S.M.); bouchamaab@ngha.med.sa (A.B.); bothinaalharbi@gmail.com (B.M.A.); nusaibahgaber@gmail.com (N.S.G.); 2 Bioinformatics and Biostatistics Department, King Abdullah International Medical Research Center, King~Saud bin Abdulaziz University for Health Sciences, MNGHA, Riyadh 11426, Saudi Arabia; rashidma@ngha.med.sa; 3 Stem Cells Unit, Department of Cellular Therapy, King Abdullah International Medical Research Center, King Saud bin Abdulaziz University for Health Sciences, MNGHA, Riyadh 11426, Saudi Arabia; khatlanita@ngha.med.sa

**Keywords:** COVID-19, SARS-CoV-2, SARS-CoV, accessory protein, ORF8, ORF8ab

## Abstract

The COVID-19 pandemic, in the first seven months, has led to more than 15 million confirmed infected cases and 600,000 deaths. SARS-CoV-2, the causative agent for COVID-19, has proved to be a great challenge for its ability to spread in asymptomatic stages and the diverse disease spectrum it has generated. This has created a challenge of unimaginable magnitude, not only affecting human health and life but also potentially generating a long-lasting socioeconomic impact. Both medical sciences and biomedical research have also been challenged, consequently leading to a large number of clinical trials and vaccine initiatives. While known proteins of pathobiological importance are targets for these therapeutic approaches, it is imperative to explore other factors of viral significance. Accessory proteins are one such trait that have diverse roles in coronavirus pathobiology. Here, we analyze certain genomic characteristics of SARS-CoV-2 accessory protein ORF8 and predict its protein features. We have further reviewed current available literature regarding its function and comparatively evaluated these and other features of ORF8 and ORF8ab, its homolog from SARS-CoV. Because coronaviruses have been infecting humans repeatedly and might continue to do so, we therefore expect this study to aid in the development of holistic understanding of these proteins. Despite low nucleotide and protein identity and differentiating genome level characteristics, there appears to be significant structural integrity and functional proximity between these proteins pointing towards their high significance. There is further need for comprehensive genomics and structural-functional studies to lead towards definitive conclusions regarding their criticality and that can eventually define their relevance to therapeutics development.

## 1. Introduction

What started in early December 2019 as a few cases of inexplicable pneumonia in Wuhan, China was officially named by World Health Organization (WHO) as Coronavirus disease 2019 (COVID-19), and the International Committee on Taxonomy of Viruses (ICTV) classified the causative agent as SARS-CoV-2 [1,2,3]. For its pan-global nature of infections and deaths, COVID-19 was declared a pandemic on 11 March 2020 [4]. More than 15 million confirmed infected cases and above of 600,000 deaths all over the world have been recorded in the first 7 months of this pandemic [5,6]. A standout characteristic of SARS-CoV-2 is the ability for asymptomatic transmission [7,8] and it is this unusual capacity to spread even during asymptomatic phases that has led to unprecedented measures to control its spread [9,10]. SARS-CoV-2 has proven to be an extraordinarily strong and lethal pathogen [11,12], impacting its primary site of infection, i.e., the nasal epithelium and lung [13,14], eliciting disproportional immune response with the potential of leading to immune dysregulation [15]. Additionally, SARS-CoV-2 has been characterized by diverse disease spectrum spread over but not limited to the gut [16], cardiovascular system [17], cutaneous system [18] and central nervous system [19], with reports of autoimmune, autoinflammatory and multisystem inflammatory syndrome in children [20,21]. 

**Figure 1:**
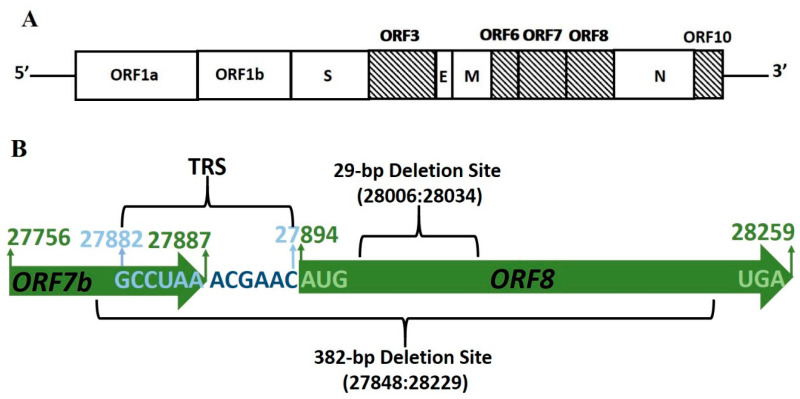
(**A**) SARS-CoV-2 genome organization: Open Reading Frames (ORFs) with a verified transcription regulatory sequence (TRS). (**B**) SARS-CoV-2 Subgenomic mRNA8 region: ORF regions and boundaries are highlighted in green. ORF8 transcription regulatory sequence (TRS) (27,882:27,894) is highlighted in blue and core TRS sequence in dark blue. Both the reported 482-bp deletion site and the predicted 29-bp deletion site are depicted. [Markings are merely representative and not up to the scale].

Similar to other coronaviruses, SARS-CoV-2 is an enveloped virus characterized by a positive-sense, single-stranded RNA genome of approximately 30 kB that codes for six major open-reading frames (ORFs); ORF1a, ORF1b, spike (S), envelope (E), membrane (M) and nucleocapsid (N) [1,2]. SARS-CoV-2 spike protein [22,23,24], main protease [25,26], helicase and RNA-dependent RNA polymerase [27] have been classified as important targets for therapeutic intervention [28,29,30,31] for their roles in receptor identification, cell entry, viral replication, and transcription. In addition to these known factors of pathobiological and therapeutic importance, a vital coronavirus-related characteristic is ‘accessory proteins’, the genes for which differ in genomic locations (Figure 1A), number and nature between coronavirus groups and have been also termed as ‘group-specific genes’ [32,33]. Although considered to be dispensable for viral replication and growth, their presence and maintenance within genomes has led to huge interest in understanding their significance to coronavirus life cycle and virulence [32,33,34]. Given the challenges that both COVID-19 and SARS-CoV-2 have posed, studies involving these pathogen-specific proteins can therefore enhance understanding of its pathobiology and translate into new opportunities and targets for the design of antiviral therapeutics. 

The SARS-CoV-2 genome is believed to harbor 6-9 accessory proteins with verified transcription regulatory sequence (TRS) identified upstream of ORFs 3, 6, 7, 8, and 10 [1] (Table 1). ORF8 is an accessory protein that is not shared by all members of subgenus sarbecovirus and it was the presence and location of ORF8 in the SARS-CoV-2 genome that led to the classification of SARS-CoV-2 genome with that of SARS-CoV [1]. SARS-CoV-2 Clade S subtype characteristics include the marker variant based on the T28144C mutation leading to L84S change in the ORF8 protein sequence [35], a positive selection that has resulted in the divergence of a separate phylogenetic group [36]. These accessory proteins offer functional flexibility to coronaviruses and accordingly are subject to alterations depending upon the condition in which they are expressed during the viral life cycle. The SARS-CoV ORF8 homolog, ORF8ab, from the closest human pathogen was accompanied by 29-nt nucleotide deletion during the mid and late phase of the epidemic, leading to two truncated and functional proteins, ORF8a and ORF8b [37]. Irrespective of the debate around the precise role of this deletion, biochemical and functional characterization of ORF8ab and its truncated counterparts has been pursued and is an ongoing process. The functional implications of SARS-CoV-2 ORF8 have already garnered attention and initial reports predict it to be an important component of immune surveillance machinery [38,39]. Keeping in mind the capacity of coronaviruses to periodically infect human populations, it becomes imperative to view them in a holistic manner and study all aspects related to their life cycle and pathogenicity. This can potentially lead to better understanding of their evolution, host-to-human transmission processes, and pathobiology that will help in the development of better combating strategies as well as guide the course of future studies. In this context, we analyze certain genome-based features of ORF8 and undertake a comparative evaluation of ORF8 and SARS-CoV ORF8ab genome stability, evolutionary origin, and protein characteristics.

## 2. ORF8 Subgenomic mRNA8 Stability

### 2.1. Genome Deletions in SARS-CoV ORF8ab

During the SARS-CoV epidemic in 2003, subgenomic mRNA8 from animal sources and early human isolates coded for a full-length ORF8ab protein. An interesting and striking feature observed in human-to-human transmission during the peak of this epidemic was a 29 nucleotide deletion (Figure S1) towards the 5′ region of ORF8, splitting it into two unequal ORFs; a smaller ORF8a and a long ORF8b that finally coded for 39 and 84 amino acid long polypeptides, respectively [40,41,42]. During late stages of this SARS epidemic, even larger deletions of 82 nt and 415 nt were identified in some virus clusters from human isolates, that led to disruption of a putative ORF9 and eliminations of ORFs 10 and 11 [42,43]. The genetic and functional importance of 29nt deletion in SARS-CoV ORF8ab has been a matter of intense debate as to whether this is a case of genomic instability or adaptive evolution and has been hypothesized to contribute to zoonotic transition and favor human adaptation [33,41,42]. The contribution to human adaptation has been attributed to the functional implications of this deletion that led to development of proteins with new functions relevant to later stages of a viral epidemic [41,42]. A contrary view to this functional implication for 29-nucleotide has been proposed, to be based in the founder effect that has permitted SARS-CoV survival despite reduced fitness and is not of a generalized role in SARS-CoV host adaptation [44]. Irrespective of the rationale behind this deletion, its existence in the SARS-CoV ORF8ab is a confirmed fact, but that has not deterred exploration of the functional significance (Section 5.1) of ORF8ab and its truncated versions ORF8a and ORF8b.

### 2.2. Genome Deletions in SARS-CoV-2

COVID-19 has spread at an extremely fast rate all over the globe in terms of both space and time. During a fast spreading and full-blown pandemic like COVID-19, there is the possibility that an extremely large number of virions are generated, and the higher the number of these virions, the higher the chances of genome-related events like mutations and deletions. Three deletion events in the SARS-CoV-2 genome have been reported so far. Deletions in the region spanning ORFs 7a and 7b were detected during a surveillance program in Arizona, USA from among the samples screened in the week of 16–19 March 2020 [45]. This 81 nucleotide deletion, leading to a 27 amino-acid in-frame deletion in one of the genomes AZ-ASU2923, maps to the putative signal peptide and first two beta strands in the protein structure [45]. A 382-nt deletion (27848:28229) has been reported from three hospitalized patients in Singapore in the genomic region spanning ORF7b and this deletion includes the transcriptional regulator sequence (TRS) of ORF8 [46]. A similar deletion was recorded in the genome of a sample (CGMH-CGU-02) isolated from a patient in Taiwan on 4 February 2020 and the patient had returned from Wuhan, China a day earlier [47]. Phylogenetic analysis of the cluster in which the 382-nucleotide deletion was reported from Singapore predicts the possibility of a single source [46] and, due to its closeness, this deletion can be considered to be of the same origin as one from Taiwan. Therefore, these cases of 382-nt deletion having their origin in isolates obtained from patients that had returned from Wuhan points towards their existence in the earlier phase of the outbreak. It is pertinent to note that the genome deletion events during viral life cycle and evolution are not usually random and are believed to play a role in helping viral genomes get rid of accumulated deleterious genome changes [48]. Because the deletions that have been reported so far from SARS-CoV-2 genomes occurred in a relatively early phase of this pandemic, therefore this cause for these deletions can be ruled out. This argument is strengthened by recent reports about the functional importance of ORF8 in immune modulation, directing more attention towards the indispensability of this protein [38,39]. Further discussions are given in the ensuing section.

### 2.3. Genomic Stability Estimation of SARS-CoV-2 ORF8

As SARS-CoV-2 has demonstrated a remarkable capacity to spread globally, consequently genomes of isolates have been sequenced all over the globe. This sequencing of a large number of isolates has has been made possible by vast progress made in next generation sequencing (NGS) via development of high-throughput platforms and other automations. Between the period between the first report of SARS-CoV-2 genome sequence in December 2019 and 30 June 2020, data for around 30,000 sequences in Global Initiative on Sharing All Influenza Data GISAID [35] and 7000 sequences in NCBI Virus [49] databases have been recorded. Keeping the enormity of this genomic data in mind, it is plausible to believe that the deletions detected and reported so far do not provide a fair estimation of the overall picture. 

Keeping this in view, we tried to run our own analysis targeting the sequences from both data repositories. For this purpose, representative sequence datasets named as NCBI Dataset (Table S1) and GISAID Dataset (Table S2) were utilized, with the NCBI Dataset containing 104 sequences and GISAID containing 177 sequences. These representative sets comprise sequences reported in the period between December 2019 and end of June 2020. To achieve a global representation, genome sequences from Africa, Asia, Europe, North America, Oceania, and South America were included, with a fair representation of the countries were high infection rates of COVID-19. 

**Figure 2:**
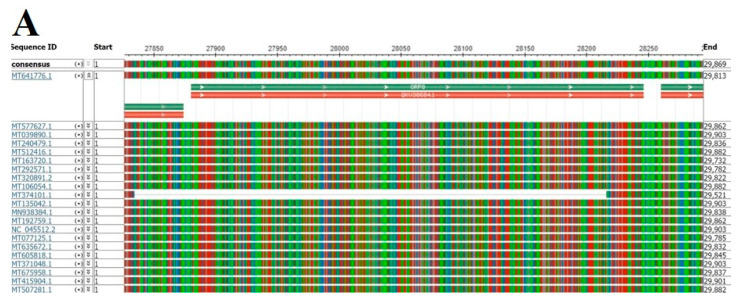

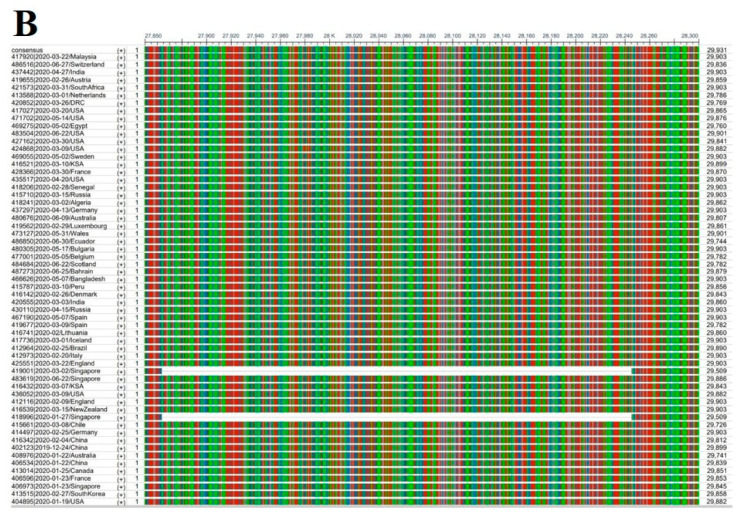
(**A**) Subgenomic mRNA8 region stability for the NCBI Dataset. Representative figure for multiple sequence alignment. An intact ORF8 coding region is detected in all sequences other than for isolate MT374101.1, which already has a known deletion at the region 27848:28229. (Details in Figure S2). (**B**) Subgenomic mRNA8 region stability for the GISAID Dataset. Representative figure for multiple sequence alignment. Deletions can be visualized in already reported isolate sequences 419,001 and 418,996 at 27865:28246. The variation in deletion location from the NCBI dataset is because of sequence alignment adjustment. The NCBI dataset numbers represent the true numbers. (Details in Figure S3).

The ORF8 gene is predicted to be 366 nucleotides (Genomic Region 27,894:28,259) in length and is preceded by a transcription regulatory sequence (TRS) on the 5′ end without any gap with the initiation codon. The reported 382-nt deletion corresponds to genomic location 27,848:28,229, and the predicted 29-nucleotide deletion site, on the basis of its similarity with ORF8ab, can be mapped to 28,006:28,034. We therefore focused on the genomic region 27,800:28,300 that covers both the full-length ORF8 as well as the reported 382-nt deletion region (Figure 1B). We focused on stability of this genomic region to identify any deletion events, detected by analyzing the multiple sequence alignment (MSA) of the two representative sequence datasets. For the NCBI Dataset, multiple sequence alignment was straightway performed utilizing the online available tools on the NCBI Virus portal. The alignment file was viewed in the NCBI Multiple Sequence Alignment Viewer 1.15.0, focusing on the region 27,800:28,300 (Figure 2A and Figure S2). Whole genome multiple sequence alignment for the GISAID Dataset was performed utilizing MUSCLE [50]. A similar approach for visualization and analysis was utilized as for the NCBI Dataset utilizing the NCBI Multiple Sequence Alignment Viewer 1.15.0 (Figure 2B and Figure S3). In these sequence datasets subjected to multiple sequence alignment analyses, we did not identify any deletions other than the ones that have already been reported, and an intact full-length ORF8, including the expected 29-nucleotide deletion region of 28,006-28,034 can be seen. A recent comparative analysis of SARS-CoV-2, SARS-CoV, and other SARSr-CoV genomes has also led to the identification of the deletion vulnerable 430bp region in the SARS-CoV-2 ORF8, identical to the SARS-COV region but without detection of any deletion [51]. The unbiased spatio-temporal coverage that is provided by our two sequence datasets, consisting of 381 representative genome sequences, affords reasonable input to estimate genome stability. It looks reasonable to conclude that, at this point, SARS-CoV-2 subgenomic mRNA8 has, to a large extent, stayed stable and potentially codes for functional full length ORF8 protein. 

The deletion events so far reported in the SARS-CoV-2 genome are limited to an extremely small number of clusters and compared to its global vertical and horizontal spread, can be presumed to be insignificant. Nonetheless, there has to be some reason behind their existence; a probable justification could be the ‘founder effect’. The founder effect is a phenomenon through which a genetically altered population of ‘founders’ is randomly selected through transmission bottlenecks and these founders retain the capacity to reproduce [52,53,54]. The founder effect, as a reason for genetic diversification, has been reported in many viruses and pathogens [55,56,57,58] including SARS-CoV [44], and, in HIV, has been an immense source of polymorphisms and evolution [55]. In a study evaluating the distribution of mutations in SARS-CoV-2 genomes, the clonal nature of mutations in different geographical regions was detected, leading to the assignment of the founder effect as the cause [59]. In the initial phases of endemic viral infections, viruses are under a constant selection pressure and certain lineages can eventually face extinction because of the competition with their more reproductively capable counterparts. However, during this initial period slightly deleterious mutations can continue to proliferate, and their existence can be expected in early stages when the virus has not fully adapted to its host environment [53]. The deletions [45,46,47] in the SARS-CoV-2 genome have been reported in very early phases of this pandemic and from three specific locations, out of which the origin for two, Taiwan and Singapore, can be traced to Wuhan. The third deletion event has been reported from a specific location, Arizona in USA. Taken together, these deletions can be attributed to genetic events particular to these clusters, and, in the absence of further reports about deletions, look like they were specific to that period of time. In addition, the number of isolates in these cases is low; three from Singapore [46] and one each from USA [45] and Taiwan [47], and with no further reports of additional deletion events from these geographic locations or anywhere else, it will be more plausible to conclude that the SARS-CoV-2 genome has not undergone any effective genomic deletion events that can have an impact on its virulence and pathogenicity. Nevertheless, in spite of the propensity for deletions in these genomic regions, exploration of the functional importance of the proteins they code for should not be compromised, especially keeping in view the ability of coronaviruses to repeatedly infect human populations with a SARS-CoV-2-like severity. 

## 3. ORF8 Protein Origin

Coronaviruses belong to Order; *Nidovirales*, Family; *Coronaviridae*, Subfamily; *Coronavirinae*, and are ordered into four genera, Alpha, Beta, Gamma, and Delta [60]. SARS-CoV-2, along with the two recent coronaviruses that infected humans, Severe Acute Respiratory Syndrome-CoV (SARS-CoV), and Middle East Respiratory Syndrome CoV (MERS-CoV), belong to the genus Betacoronavirus that is further classified into lineages A to D [42,61,62]. Bats are recognized as an important reservoir of several emerging viruses that include alphacoronaviruses and betacoronavirus lineages B, C, and D. Approximately 200 coronaviruses with bat origin have been identified with these coronaviruses regarded as the major source of genes for mammalian coronaviruses [63,64,65]. These viruses, being RNA viruses, are known to be prolific evolvers for their high rates of replication and mutation, coming from infidelity of their RNA-dependent RNA polymerase (RdRp), and that allows their fast movement through sequence space. In addition to this capacity to generate variations through mutations, these viruses also hold the capacity to exchange genetic material. Exchange of genetic material can occur through two distinct but not completely exclusive approaches; reassortment and recombination [48]. Reassortment is a trait associated with multipartite viruses like influenza A and involves the swapping of discrete RNA molecule(s) from the segmented viral genome. Recombination, on other hand, that can occur both in segmented and unsegmented viruses involves the introduction of ‘donor’ nucleotide sequence into an ‘acceptor’ RNA molecule that can potentially contain genetic information from multiple sources. 

### 3.1. SARS-CoV ORF8ab Bat Origin

SARS-CoV ORF8ab is believed to have originated from potential recombination events between betacoronaviruses from greater horse-shoe bats *Rhinolophus ferrumequinum* (SARSr-Rf-BatCoV) and Chinese horseshoe bats *Rhinolophus sinicus* (SARSr-Rs-BatCoV) [62]. SARSr-Rs-BatCoV and SARSr-Rf-BatCoV both share a remarkably high genome identity, 95% and 93%, respectively, with human/civet coronaviruses. ORF8ab amino acid identities do not reflect the same picture as is observed in genome identities; only SARSr-Rf-BatCoVs share 80.4–81.3% identity, while SARSr-Rs-BatCoVs share only 32.2–33%. On the basis of high genome identity between SARSr-Rf-BatCoVs, SARSr-Rs-BatCoVs and human/civet coronaviruses, and high identity in ORF8ab between SARSr-Rf-BatCoVs and human/civet coronaviruses, the authors came to the conclusion that that the ancestor of civet SARSrCoVs acquired its ORF8ab from SARSr-Rf-BatCoVs through its recombination with SARSr-Rs-BatCoVs. This was verified by the identification of potential recombination sites between SARSr-Rf-BatCoVs and SARSr-Rs-BatCoVs around the ORF8ab region, leading to the belief that civet SARSr-CoV SZ3 evolved with its ORF8ab acquired from SARSr-Rf-BatCoVs [62]. In a comprehensive study exploring coronaviruses from bats and analyzing their genetic variations with human/civet coronaviruses, phylogenetic clustering of SARSr-Rf-BatCoV ORF8ab with human/civet SARSr-CoVs was observed [66]. The bat origin of ORF8ab specifically from *Rhinolophus sinicus* has also been reported elsewhere through the identification of ORF8ab homologs in “SARS-like CoVs” (SL-CoVs) in these bats [67]. Therefore, it can be concluded with confidence that SARS-CoV ORF8ab has primarily originated from bat coronaviruses [67].

**Figure 3:**
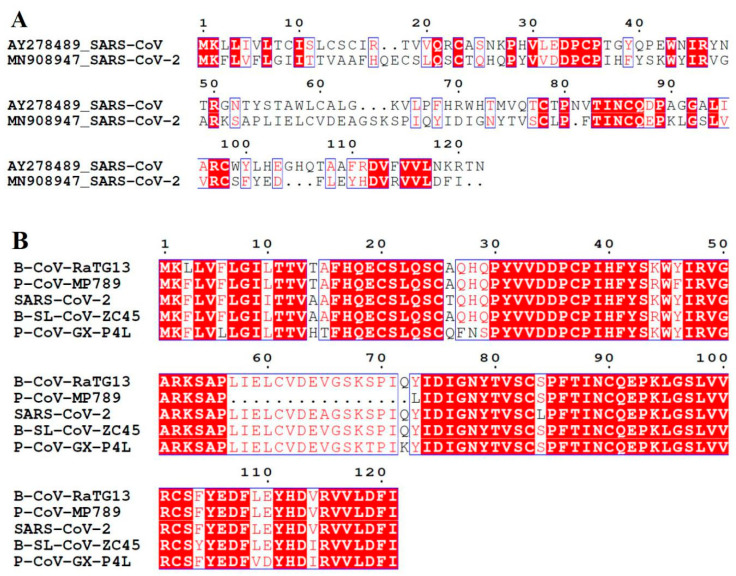
Protein sequence alignments of human SARS-CoV-2 ORF8. (**A**) Alignment with ORF8ab (AAP51236.1) of SARS-CoV. (**B**) Multiple sequence alignment with the high identity (80–100%) proteins identified from blastp search against the NCBI non-redundant protein sequences (nr) database. Sequences have been labelled according to isolate, and predicted proteins corresponding to these isolates are non-structural protein (NS8) for Bat-CoV-RaTG13, a hypothetical protein for Bat-SL-CoVZC45, and ORF8 for two pangolin isolates.

SARS-CoV ORF8ab and SARS-CoV-ORF8 share low nucleotide (26%) and protein (20%) identities (Figure 3A), thus making straightforward interpretations about evolutionary and phylogenetic relationships difficult. Likewise, this molecular identity makes it important to analyze the origin of SARS-CoV-2 ORF8 protein. 

### 3.2. SARS-CoV-2 ORF8 Homologous Proteins

As there is no published literature regarding the ORF8 origin or its homologous proteins, we therefore searched multiple genome and protein data repositories in pursuit of identifying its homologs. Using BLAST [68] searches, four proteins with identities ranging between 80 and 100% (Figure 3B) were identified from the NCBI non-redundant protein sequences (nr) database, UniProtKB and Protein Databank (PDB). These four proteins were shortlisted because they provided stringency in terms of a 100% coverage against input SARS-CoV-2 ORF8 amino acid sequence (Table 2). The first two high identity homologs are from bat coronaviruses Bat-CoV-RaTG13 and Bat-SL-CoVZC45, among them there is remarkably high homology (95%) with Bat-CoV-RaTG13 non-structural protein 8 (NS8). This high homology with RaTG13 non-structural protein 8 is not surprising because it is the closest relative of SARS-CoV-2 with an overall nucleotide identity of around 93–95% [1,2]. Protein level identities between other SARS-CoV-2 and RaTG13 proteins have been recorded, for example, the SARS-CoV-2 S gene coding for receptor binding spike protein shares 93.1% nucleotide and 98% protein identity and this potentially affords some differentiating characteristics to SARS-CoV-2 spike protein [2,69]. Despite this close homology between bat and human infecting coronaviruses and the knowledge about the bat source of coronaviruses, direct bat-to-human transmission has always been ruled out. In the case of SARS [41] and MERS [61], these transmissions are believed to happen through zoonotic routes of civets and dromedary camels, respectively. The fact that these animals play a role, either as intermediate or amplifying host, has led to speculations that intense genome modification events within bats and intermediate hosts contribute to animal-to-human transmission and potentially to the virulence of these viruses [66,70]. Pangolins have received considerable attention as an intermediate host for SARS-CoV-2 infection for the reasons that coronaviruses isolated from pangolins have significant genome level identity with the SARS-CoV-2 genome as well as with bat coronaviruses that have high similarity with SARS-CoV-2 [71,72,73,74]. Therefore, it is not a surprise that the other two high identity homologs of ORF8 detected in our searches belong to pangolin coronaviruses; Pangolin-CoV-GX-P4L shares 81% identity while Pangolin-CoV-MP789 is 86% identical (Table 2). High amino acid identities with pangolin coronavirus homologs have also been reported in cases of other SARS-CoV-2 proteins. A recently reported Malayan pangolin coronavirus isolate shares amino acid identities of 100% in envelope (E), 98.2% in main proteinase (M), 96.7% in nucleocapsid (N) and 90.4% in spike (S) proteins, with the receptor-binding domains (RBD) of spike protein being almost identical, carrying only a single amino-acid difference [71,72,75]. Thus, ORF8 behavior in terms of its genomic and protein identity is like its other counterparts from SARS-CoV-2 that show a strong identity with both bat and pangolin coronaviruses. 

### 3.3. SARS-CoV-2 ORF8 Evolutionary Pathway

Genome level identities among SARS-CoV-2, bat and pangolin isolates identified in our ORF8 protein homolog searches are considerably high, varying between 80 and 95% (Table 3). Some Pangolin coronavirus genomes have remarkably high homology (around 90%) with the SARS-CoV-2 genome [71]; a recently identified Malayan pangolin, Pangolin-CoV-2020, shares 90.32% [73], Pangolin-CoV 91.02% [72], Pan_SL_CoV_GD 91.2% [76], and Pan_SL_CoV_GX 85.40% [76] identity with SARS-CoV-2. Therefore, a question arises as to whether ORF8 has descended directly from bat or pangolin coronavirus sources or some genome modification events have contributed towards its evolution. The coronavirus ORF8 region, along with nsp3, ORF3 and S, are considered to be among the rapidly evolving regions being flanked by recombination-prone sequences [36,62,67], thus augmenting the possible contribution of recombination in coronavirus and coronavirus element evolution. A recent publication has also reported the presence of recombination breakpoints around ORF8, raising the possibility of modular recombination occurring at both ends of ORF8 that are characterized by near identical nucleic acid sequences among SARS-CoVs and some bat CoVs [76]. They also observed that phylogenetic analysis in SARS-CoVs and analyzed bat coronaviruses based on the region around ORF8 led to their distinct and divergent clustering, pointing towards recombination around this region. In the initial phylogenetic analysis performed during its identification, the first SARS-CoV-2 isolate clustered with members of the subgenus Sarbecovirus but changed the topological position with respect to the gene used for phylogenetic estimation, leading to a conclusion that recombination has played a role in the evolution of these coronaviruses [1].

**Figure 4:**
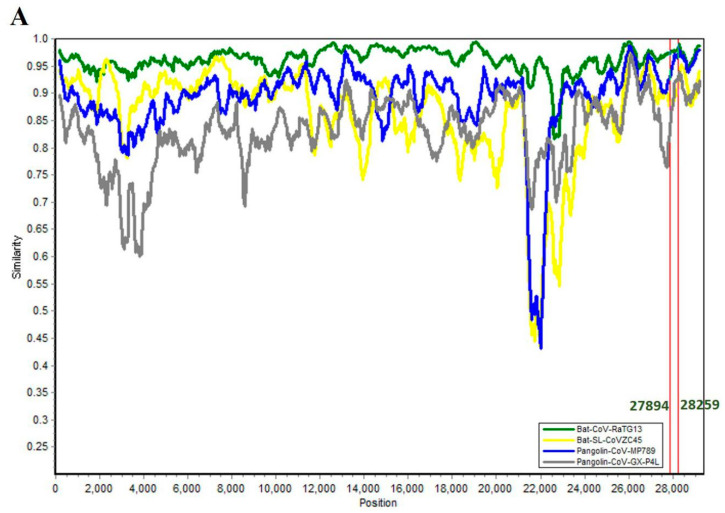

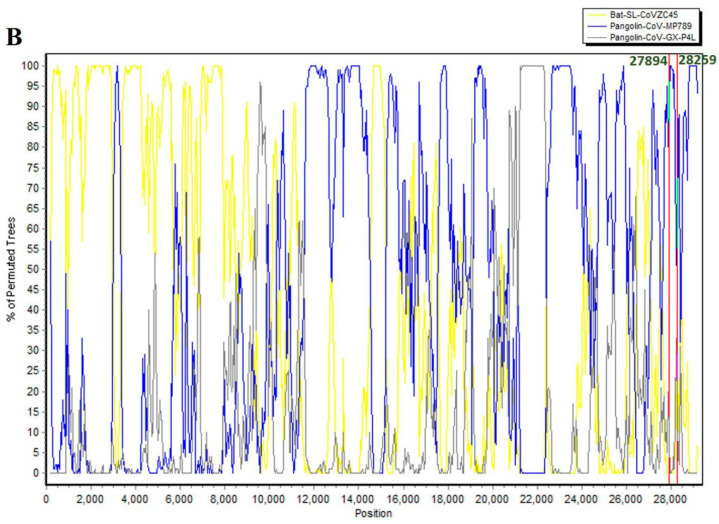
(**A**) SimPlot genetic similarity plot between SARS-CoV-2 (MN908947.3) and sequences for bat and pangolin isolates for which ORF8 homologs show a high protein identity. (**B**) BootScanning was conducted with Simplot version 3.5.1 using a 400–base pair (bp) window at a 50-bp step and the Kimura two-parameter model on a nucleotide alignment, generated with ClustalW. The highlighted region 27894-28259 corresponds to genomic coordinates of ORF8.

With these facts and the close molecular identities between these ORF8 homologs harboring coronavirus isolates from bat and pangolin sources in mind, we tried to understand role of recombination in the origin of SARS-CoV-2 ORF8. Recombination analysis involving the SARS-CoV-2 genome and four high identity genomes (Table 3) was performed using Simplot 3.5.15 [77] and RDP4 [78]. Bat-CoV-RaTG13 has highest sequence similarity with SARS-CoV-2 and also shares a close phylogenetic relationship that is visible in the SimPlot genetic similarity plot, where the RaTG13 plot can be seen visibly separate from the Bat-SL-CoVZC45, Pangolin-CoV-GX-789, and Pangolin-CoV-GX-P4L profiles (Figure 4A). Identification of potential recombinant regions was accomplished by sliding a 400-base pair (bp) window at a 50-bp step across the alignment using the Kimura (2-parameter) model. When SARS-CoV-2 was used as a query with Bat-SL-CoVZC45, Pangolin-CoV-GX-789, and Pangolin-CoV-GX-P4L, several potential breakpoints could be identified (Figure 4B), but there was not a clear cut recombination crossover point around the subgenomic region coding for ORF8 (27,894:28,259). Utilizing RDP4 for analysis, a total of 23 potential recombination cross-over points could be detected with the genomes involving different parental relations. Out of these 23, seven recombination events (Table 4) had the capacity to bring changes in the SARS-CoV-2 genome, while SARS-CoV-2 involvement as a parent (Table 5) could be identified in 15 events, nine of which involved SARS-CoV-2 as the major parent. The results from both analyses suggest role of cross-species recombination in SARS-CoV-2 evolution, something that has been observed before also [76,79]. Similar to the SimPlot recombination analysis, none of the events identified in RDP4 analysis covered the ORF8 coding subgenomic region. These results are somewhat surprising in the context of what is reported and expected about recombination around ORF8 (Starting lines of this paragraph), especially in view of the ORF8 protein identities with their homologs from bat and pangolin coronaviruses and the genome identities between these viruses. A simple and a plausible explanation could be that this analysis needs to be conducted with a larger sequence dataset that creates enough input for identifying and scoring these recombination events. At the same time, it needs to be kept under consideration that recombination is not the sole factor that contributes to evolution of coronavirus proteins. As an example, the high amino acid similarity of SARS-CoV-2 spike protein receptor binding domain (RBD) with that of coronaviruses isolated from Pangolins in Guangdong, China has been proposed to be selectively mediated by convergent evolution [74]. Two amino acid substitutions, 436Y and 427N, in the RBD of SARS-CoV-2 spike protein are also present in SARS-CoV but not in the highly homologous spike protein of RaTG13, a potential adaptive convergent evolution in Sarbecovirus infecting humans [80]. Although it might be too early to speculate, one is tempted to think about role of adaptive convergence in human-infecting SARS-CoV ORF8s. The two proteins share low protein identity (20%), but in spite of that there is conservation of certain protein features (Section 4) and, consequently, functional characteristics (Section 5) among them. Even though SARS-CoV ORF8ab was accompanied by 29-nt deletion, the truncated versions still retained their functions, and, in fact, functional diversity was speculated to be one of the reasons behind truncation. On the contrary, as we saw in previous section that SARS-CoV-2 ORF8 has not undergone any significantly measurable deletion events, so its function as a full-length protein might be more important to its pathogenicity. Despite this potential variation in the functional relevance of ORF8 to two pathogens, there is a significant overlap between them both in terms of the functions and mechanisms behind them. A role for parallel or convergent changes is to create signals for adaptive evolution because it is highly unlikely that significant and complex characters can originate through multiple chances [81]. At a deeper molecular level, a significant commonality between these proteins can only be identified from information about their structures, which is lacking this time. In addition to the established importance of protein structure, it is also important to help trace the origin and evolutionary pathway of these proteins. Therefore, structural biology efforts must be made in earnest and should involve SARS-CoV-2 ORF8, its homologs from reservoir, i.e., bat coronaviruses, and from the intermediate host, i.e., pangolin coronaviruses. The exploration of pangolin genomes is also being pursued vigorously and all this together will facilitate large studies that can help develop an understanding of the genetic processes involved in their evolution and the unravelling of mechanisms behind the acquirement of traits and functions that facilitate their animal-to-human transmission and virulence. 

## 4. Conserved Features of SARS-CoV-2 ORF8 and SARS-CoV ORF8ab Proteins

Knowledge of protein tertiary structure is critical to the understanding of their functions as well as to the understanding of their mechanisms of action. Atomic resolution structural details lead to molecular level information about unique protein features that can be exploited in therapeutics development. Keeping in mind the importance of protein structures, substantial efforts based in structural biology and computational protein modelling have been dedicated towards obtaining structural information about SARS-CoV-2 proteins. ORF8 has proved to be an intractable protein to computational biology-based modeling approaches as templated-based homology modeling approaches are not applicable, nor have machine-learning based tools like I-TASSER [82], AlphaFold [83] or even a newly developed approach [84] specifically for SARS-CoV-2 proteins met with success. A comparative analysis of different modeling approaches applied to SARS-CoV-2 proteins concluded an unreliability and inefficacy of protein modelling approaches in certain SARS-CoV-2 proteins including ORF8 [84]. Computational structural biology approaches at this stage might not be a reliable approach to study ORF8, emphasizing the need for such studies and, in the interim, making it more prudent to rely on methods like knowledge-based ones. We utilized web-based resources that together contain a diverse suite of predictive tools and can be salvaged to reliably deduce information about protein structural and functional features. SARS-CoV ORF8ab has been extensively characterized for its biochemical features, although atomic structural level information is still not available. Comparison between information generated about ORF8 here with the available knowledge about ORF8ab can be considered to be reliable enough to build a good understanding of protein features and will guide in the design of deeper and more comprehensive structure-functions studies. 

### 4.1. Endoplasmic Reticulum Residence

The full-length SARS-CoV ORF8ab was identified as a stable endoplasmic reticulum resident protein by virtue of its cleavable N-terminal signal sequence that directs its transport to ER [37]. The EGFP tagged 8ab and 8a fusion proteins displayed a quite similar reticular pattern, while the 8b-EGFP fusion protein was found to be distributed all over the cells. The difference in localization is potentially due to the loss of N-terminal signal peptide as a result of 29-nt nucleotide deletion. The ER localization of ORF8ab was further validated by fluorescence colocalization studies with ER marker calreticulin and ER residence by pulse-chase experiments. Partly contrary to this, ORF8ab was found to be membrane bound identified through detergent resistance and microsome association behavior similar to calnexin, an ER-resident membrane protein [85]. Incidentally, similar to a previously mentioned study [37], in these experiments, calreticulin was also used as control to identify ER luminal behavior. The differences in outcomes from the two studies can potentially be either due to difference in the cellular systems used (OST7-1 and HeLa Cells) or the method of evaluation (Immunofluorescence and Detergent Resistance). Irrespective of whether ORF8ab is a membrane bound or a soluble protein, its ER residence can be considered with conformity. 

Sequence analysis of SARS-CoV-2 ORF8 predicts an N-terminal signal peptide located within amino acids 2-16, with the 5-13 stretch having high hydrophobicity, a feature resonant with the 6-15 hydrophobic core signal sequence characteristics [86,87,88]. Endoplasmic reticulum, in contrast to cytosol and other cellular organelles, has a predominantly oxidative environment that provides an essential ecosystem for oxidation, protein folding and protein quality control processes [89,90]. In terms of its ion concentration and redox potential, the ER ecosystem matches very well with the extracellular environment where secreted and surface proteins work [90,91,92]. One of the important functions that endoplasmic reticulum performs by virtue of its oxidative environment is the introduction of intra- or intermolecular disulfide bonds between unpaired cysteine residues of polypeptides [89,93]. The cysteine content of the secretory pathway and mammalian extracellular proteins is higher than cytosolic proteins, correlating with the requirement for oxidative protein quality systems afforded by endoplasmic reticulum [89]. SARS-CoV-ORF8ab protein is characterized by the presence of 10 cysteine residues and exists as disulfide-linked homomultimeric complexes in endoplasmic reticulum [37]. ORF8 has seven cysteine residues at positions 20, 25, 37, 61, 83, 90 and 102 and can be expected to engage in disulfide bridge formation with two separate predictions forecasting three disulfide bonds. Keeping these facts in consideration, it can be at reasonably concluded that ORF8, like SARS-CoV ORF8ab, is an ER resident protein with similarities spanning the presence of the N-terminal signal peptide sequence and the potential to form disulfide bonds. That ORF8 is an ER resident protein can be gauged from the fact that its host interactome identified in HEK-293T/17 cells significantly consists of endoplasmic reticulum resident proteins involved in pathways like protein quality control [28]. 

### 4.2. Conversed Glycosylation Site

Glycosylation is an important step in protein maturation and involves the attachment of sugar moieties that contributes to both the stability and solubility of a protein. SARS-CoV ORF8ab is characterized by the presence of an N-glycosylation site structured around asparagine 81, with overall motif organization as Asn-Val-Thr [37,94]. N-linked glycosylation involves oligosaccharyl transferase (OST) complex-facilitated attachment of core Glc_3_Man_9_GlcNAc_2_ (3 glucoses, 9 mannoses and 2 N-acetylglucosamines) to the amino acid nitrogen atom, which is usually N4 of asparagine [95,96]. This interaction happens at typical sequence motifs with a design Asn-X-Thr/Ser/Cys, in the decreasing order of probability between Thr, Ser, and Cys and where X is a residue other than proline [97,98]. 

**Figure 5:**
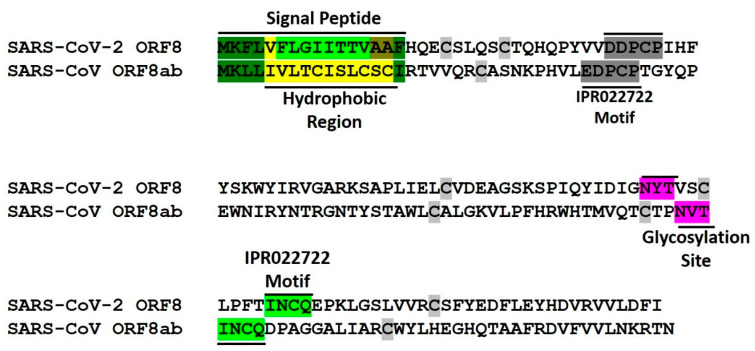
Protein characteristics of SARS-CoV-2 ORF8 and SARS-CoV-ORF8ab. Sequences are aligned to depict precise positions of sequence-based features and with respect to one another. INCQ and EDPCP are two conserved motifs in proteins belonging to InterPro family IPR022722. Cysteine residues have been highlighted in grey other than at positions that fall within other conserved sites.

At least one N-glycosylation motif can be very well identified in the ORF8 sequence with asparagine 78 being the potential site of glycosylation and the motif as Asn-Tyr-Thr (Figure 5). The relatively similar locations, ORF8ab glycosylation’s site position at Asn-81 and ORF8 glycosylation’s site at Asn-78, can be construed as an indicator of potential structural conservation between these proteins. Protein glycosylation is an important aspect of viral biology and pathogenicity and is a known fact that enveloped viruses like coronaviruses use the host cell glycosylation machinery very well. Although viral protein glycosylation is relevant in functions like viral attachment, cell entry, assembly and exit, and viral spread, the most important function that has been assigned to glycosylation is its role in the evasion of the host immune system [99,100,101]. Cryo-EM and mass spectrometric analysis of SARS-CoV-2 spike protein has led to the identification of N-linked glycan occlusion of receptor binding sites, a feature observed in other viral glycoproteins with the purpose of concealing a structurally conserved and functionally important region of the spike protein [102]. Because ORF8 is not in all probability involved in functions related to cell entry or fusion, we therefore speculate that protein glycosylation has a role in its folding and stability function over a role in circumventing the immune system. In fact, a structure stabilizing role has been detected for glycosylation in SARS-CoV ORF8ab, where the glycosylation-defective 8ab mutant was found to be unstable, similar to truncated protein 8b that has lost its glycosylation site by virtue of 29-nt deletion [94].

A unified picture (Table 6) that can be drawn of ORF8 and ORF8ab is that they are endoplasmic resident proteins transported there by virtue of an N-terminal hydrophobic signal peptide. They are further characterized by the presence of a conserved N-glycosylation site potentially playing a role in their stabilization. The presence of cysteine residues with the capacity and potential role to form disulfide bonds points towards higher levels of structural organization and a greater functional role in the viral pathobiology. 

## 5. Functional Landscape of SARS-CoV-2 ORF8 and SARS-CoV ORF8ab

### 5.1. SARS-CoV ORF8ab, ORF8a and ORF8b Functions

Despite confirmed deletion of a 29-nucleotide region, the functional importance of three translation products of SARS-CoV sub-genomic mRNA8, full-length ORF8ab, ORF8a and ORF8b has been a matter of significant interest. Multiple studies have been conducted to identify their roles in pathogenicity modulation, virus growth, replication, and host interactions. 

#### 5.1.1. Viral Replication

One of the highly studied and hotly contested functional importance of SARS-CoV ORF8ab and its truncated versions is their role in the replication of results from different studies, pointing towards both their importance and lack of a role in viral replication. While some studies have reported a role for truncated ORFs 8a and 8b in viral replication [103,104], an intact ORF8ab [105] and ORF8b [106] were not found to impact replication efficiency, duration of replication, or SARS-CoV pathology. In a recent comprehensive study evaluating the impact of 29nt deletion on the replication properties of SARS-CoV in relevant models of human respiratory tract infection and other organisms that included primate, Rhinolophus bat, cotton rat, goat and sheep cells, a 23-fold decrease in replication was observed [44]. The authors further assign the observed discrepancy (3-fold reduction) [105] in fold decrease in replication to the high MOIs used in this study, which are not close to doses at which natural infections occur. However, they have not dealt with the mechanistic details of this ORF8ab-mediated viral inhibition, but there have been other reports that proposed ORF8b-mediated inhibition of replication through the inhibition of the envelope (E) protein of SARS-CoV. The interaction of 8b with other SARS-CoV proteins like membrane (M), ORFs 3a and 7a, in addition to envelope (E), has been detected [107]. Reduction in E protein expression as a consequence of 8b overexpression was identified as acting through a post-translational mechanism and not through a mechanism that can lead to reduction in E gene expression. This down-regulation of E by 8b in a later study was found to play a negative role in viral replication involving an ubiquitin-independent proteasome pathway [106]. The ubiquitin-independent nature of this down regulation was deduced through mutational analysis of 8b lysine residues that were found to be critical only for monoubiquitination. The ubiquitination potential of ORF8ab and 8b has been reported, with these proteins reported to be post-translationally modified by ubiquitin with the ability to form both mono- and polyubiquitinated species [94]. 

The inhibition of replication might seem counter-intuitive to the requirement for viral proliferation, but one of the purposes of viral evolution is the attainment of balance between effective transmission and evasion of host immune response. Consequently, the viral replication inhibitory effect of ORF8b has been ascribed to a replication modulation function with the 29-nt deletion contributing to fine-tuning of viral-replication [107], because ORF8a [103] has been reported to work contrary to ORF8b in a pro-replication role. Nonetheless, a conclusion that can be drawn is that subgenomic mRNA8 of SARS-CoV leads to translation products that play an essential role in viral replication and consequently in its pathogenicity.

#### 5.1.2. Immune Modulation

The other important function of ORF8ab, or its truncated counterparts that has garnered interest is their role in immune modulation. This is not surprising as one of the primary strategies during viral infection involves overcoming host immune response. That SARS-CoV pathogenesis is to some extent controlled by immune signaling; this was deduced from the association of SARS-CoV with anomalous cytokine and chemokine responses and the expression of interferon stimulated genes (ISGs) in infected patients [108,109]. Multiple models of SARS-CoV infection have led to the identification of critical components of innate signaling pathways that have a protective role against SARS-CoV disease [108], and thus a viral response to circumvent this challenge is expected. The roles of non-structural proteins like nsp1, nsp7, nsp14, nsp15, and nsp16, structural proteins like membrane, and nucleocapsid and accessory proteins like ORF3b, and ORF6 have been comprehensively elucidated [33,108]. ORF8ab and ORF8b were reported to work as IFN antagonists having a role in the evasion of the immune surveillance system by acting at one of the initial steps of the INF-β signaling cascade. Both ORF8ab and its truncated counterpart were found to physically interact with interferon regulatory factor 3 (IRF3) and lead to its degradation in a ubiquitin proteasome-dependent manner [109]. Ubiquitin proteasome system (UPS)-mediated proteasomal degradation is a common strategy employed by many viruses in immune surveillance evasion by targeting host immune signaling and adaptor molecules like those involved in type I interferon (IFN) response and MHC class I antigen presentation [110]. Thus, one of the functions SARS-CoV subgenomic mRNA8 products are involved is in immune evasion through the hijacking of the host ubiquitin proteasome system (UPS). Another known mechanism by which a translation product of subgenomic mRNA8 has been reported to be involved in immune evasion is through the formation of insoluble protein aggregates. The existence of protein aggregates post viral infections has been attributed to their usage as replication scaffolds and a protective mechanism against host-induced protein degradation [111]. ORF8b forms insoluble intracellular aggregates in macrophages, leading to the activation of NLRP3 inflammasomes. This leads to the activation of transcription factor EB (TFEB), the master regulator of autophagy and lysosome machinery and is facilitated by direct interaction of ORF8b with the leucine-rich repeat domain of NLRP3 [112]. These nucleotide-binding oligomerization domain (NOD)-like receptors (NLRs) are involved in the activation of diverse signaling pathways through factors that lead to the production of immune effectors like type I interferons (IFNs), interleukin-1β (IL-1β) and IL-18, while some members have been shown to inhibit NF-κB and type I IFN-mediated signaling pathways, pointing towards the role of NLRs in the control of innate immunity [113,114]. Thus, like other viruses, SARS-CoV involves strategies that allow it to evade the immune surveillance system, and ORF8ab and OFR8b form as essential component of this process.

#### 5.1.3. Unfolded Protein Response (UPR) Modulation

A process level function that has been reported for ORF8ab is the regulation of unfolded protein response (UPR). This happens through upregulation of the synthesis of endogenous endoplasmic reticulum (ER) resident chaperones. Ectopic ORF8ab-induced expression was observed at the promoter of glucose-regulated protein 78 (GRP78), one of the best characterized ER chaperones, and ORF8ab also led to the upregulation of other ER-resident chaperones like GRP94 and CRT [85]. GRP78, also known as immunoglobulin heavy-chain binding protein (BiP), is an ER master controller that through interactions with PKR-like ER kinase (PERK), activates transcription factor 6 (ATF6) and the ER transmembrane protein kinase/endoribonuclease (IRE1) to ensure the differential regulation of ER stress required during viral pathogenesis and replication [115]. ORF8ab-mediated transcriptional activation of GRP78 was found through activation of ATF6, which binds to ER stress response element (ERSE) promoter elements of ER chaperones and is an essential requirement for their transcriptional activation. This ATF6 activation by ORF8ab occurs through direct interaction between the two proteins in ER lumen, leading to the movement of ATF6 into Golgi, the induction of its proteolytic cleavage and the forward release of its N-terminal fragment to the nucleus for activation of expression of the target genes of ER stress.

A common theme in the functionality of ORF8ab and its truncated counterparts is the capacity to bind proteins, be they viral proteins like membrane (M), ORF3a, ORF7a, and envelope (E) [107], or host proteins like interferon regulatory factor 3 (IRF3) [109]. These physical interactions with proteins lead to their degradation and eventual down-regulation, thus impacting the processes they are involved in. The mechanistic spectrum through which these functions are achieved include apoptosis [103], proteasomal degradation [106], autophagy [112], and unfolded protein response [85]. Therefore, an overall picture can be drawn of ORF8ab, ORF8a, and ORF8b as components of protein homeostasis. To firmly establish the existence and role of these functions, more coordinated studies need to be undertaken that remove the potential variation and bias created from the choice of cellular systems and experimental methodologies.

### 5.2. SARS-CoV-2 ORF8 Functions

In spite of the short duration since the start of the COVID-19 pandemic, SARS-CoV-2 ORF8 has garnered reasonable attention, but so far only few studies related to its function have been conducted.

#### 5.2.1. Immune Modulation

Overexpression of ORF8 led to significant down-regulation of MHC-I (HLA-A2) in 293T cells, human fetal colon cell line FHC, human bronchial epithelial cell line HBE, and human liver cell line Huh7 [38]. ORF8-mediated downregulation of MHC-I in 293T was ascribed to the lysosomal degradation pathway with ORF8 binding of MHC-I leading to protection against the cytotoxic T-lymphocyte (CTL)-mediated lysis of SARS-CoV-2-infected cells. ORF8 strongly colocalized with Calnexin and LAMP1, ER and lysosomal proteins, respectively, alluding towards MHC-1 degradation in endoplasmic reticulum via trafficking from ER to lysosome. The endoplasmic reticulum-associated degradation (ERAD) pathway and ubiquitination-mediated degradation of MHC-I were ruled out from knock down experiments involving ERAD pathway proteins and the absence of significant ubiquitination of MHC-I [38]. While this potentially offers a unique mechanism for the evasion of immune surveillance and a probable source of therapeutic intervention, the authors of this study as well as a follow up commentary warrant the need for further detailed mechanistic studies [38,116]. In another study analyzing the SARS-CoV-2 response to host innate immunity, ORF6, ORF8, and nucleocapsid protein were identified as potential inhibitors of the type I interferon signaling pathway acting through inhibition at promoter sites and also by inhibiting interferon-stimulated response element (ISRE), but with a low activity for ORF8 [39]. A unique COVID-19 disease phenotype has been described from severe and critical patients that consists of a highly impaired interferon (IFN) type I response that is characterized by low IFN-α production and activity and no IFN-β, which was associated with persistent blood viral load [117]. Thus, ORF8 is apparently involved in one of the most important pathways of viral pathobiology and might be the reason that the subgenomic mRNA8 region of SARS-CoV-2 has not undergone significant deletions. Although SARS-CoV ORF8ab and 8b were also reported to have similar function achieved by the inhibition of interferon regulatory factor 3 (IRF3) [109] through a protein–protein interaction, a protein–DNA interaction can be speculated in the case of SARS-CoV-2 ORF8-mediated interferon signaling pathway inhibition [38]. But this will be too premature to conclude and will therefore need validation and further studies that can uncover the mechanistic aspects. Nevertheless, there appears to be a reasonably high functional overlap between the two homologs.

#### 5.2.2. Endoplasmic Reticulum Protein Quality Control

There is another report that, despite not being intended to study ORF8 functions directly, still provides some clues about their role. The purpose of this study was to evaluate the SARS-CoV-2 human protein–protein interactions within human cells (HEK293T/17) with a goal of identifying any potential therapeutic targets [28]. In this interactome analysis, ORF8 interactions were detected with proteins involved in endoplasmic reticulum protein quality control, glycosylation, extra-cellular matrix organization (ECM), and glycosaminoglycan synthesis. With a rationale that host–virus interface processes and host targets are less prone to mutations, and can therefore be subject to therapeutic intervention, IVHR-19029, a known potent endoplasmic reticulum α-glucosidases I and II inhibitor was identified as a molecule of interest that can be targeted to ER Protein Quality Control for its member interactions with ORF8 [28]. This molecule has also been identified as a potent antiviral in other viral infections like hemorrhagic fever viruses [118,119].

Reading these results together with the role in MHC I downregulation and inhibition of interferon signaling, initial inferences can be made that SARS-CoV-2 functions involve impacting different viral or host–pathogen processes through interactions with other macromolecules like proteins and DNA. This macromolecule-interacting nature of ORF8 at the protein level matches very well with that of SARS-CoV ORF8ab, which interacts with both viral [107] and host proteins [109]. The development of a comprehensive and meaningful understanding of ORF8 necessitates the requirement for large-scale system level studies like proteomics conducted in a systematic and temporal manner that can help ascertain its stage-specific role and analyze its impact on other viral and cellular processes.

## 6. Conclusions

SARS-CoV-2 is the seventh coronavirus to infect humans that primarily causes respiratory illnesses; NL-63, 229E, HKU1 and OC43 lead to mild illness, MERS-CoV and SARS-CoV cause more serious forms of disease [120], while SARS-CoV-2 has proven to be a different kind of challenge. Bats are the major hosts for coronaviruses and by virtue of being RNA viruses, they hold a remarkable capacity to mutate and potentially lead to the existence of a large number of viral variants. This is exacerbated by their propensity for inter-species zoonotic transitions that potentially contribute to further complexity in their genomes. All these factors make them potent and formidable pathogens with the capacity to infect and challenge for a long time; therefore, they are a considerable public health concern. Keeping this in consideration, there need to be concerted efforts towards developing an understanding of their pathobiology with the aim of identifying new targets, pathways, and approaches for therapeutics development. Most of the current therapeutic strategies rely upon known proteins of pathobiological importance like spike protein, main protease, helicase and RNA-dependent RNA polymerase. The way COVID-19 is presenting itself has necessitated approaches like drug-repurposing, and a relentless pursuit towards vaccine development. Keeping all these factors under consideration, it will be prudent to expand the base towards developing our understanding of coronavirus biology and pathogenicity with the hope that it can lead to the development of new avenues for therapeutics development.

We have attempted here to comparatively study one of the accessory proteins pertaining to subgenomic mRNA8 from two recent human-infecting coronavirus pathogens (Table 6). The two proteins have remarkably low identity at the nucleotide (26%) and protein level (20%), probably due to their different origins; ORF8 looks to have originated from both bat and pangolin as compared to SARS-CoV ORF8ab, which is of bat origin. At the protein level, both proteins are characterized by the presence of an N-terminal hydrophobic signal peptide, a conserved N-glycosylation site, and enough cysteine residues with the potential to form disulfide bonds, drawing their picture as structurally stable potential ER-resident proteins. There is functional overlap between these proteins with involvement in immune modulation, which is probably accomplished through involvement in protein quality control. The purported functioning of ORF8 through endoplasmic reticulum-associated degradation (ERAD) [28,38] and SARS-CoV ORF8ab in unfolded protein response (UPR) [85] deal with two different aspects of the ER protein quality process, but the two processes are not completely independent [121,122], thus pointing to some overall process-driven role for two less identical homologs. It could possibly be that these proteins belong to a coronavirus-specific protein family. To firmly establish this or their role in coronavirus pathobiology, it will be necessary to conduct further comprehensive studies that involve approaches based in areas like genomics and evolution, systems-biology, proteomics, and structural biology. We believe that these studies should not be limited to known human pathogens only but should target other zoonotic viruses as well. Structure studies have an added advantage in that they can identify unique domains or other structural motifs and can provide insights into mechanistic details that can be exploited in the development of anti-viral therapies.

## Figures and Tables

**Table 1:** SARS-CoV-2 Accessory proteins. Genomic coordinates of ORFs along with their gene and protein length. The transcription regulatory sequence (TRS) location for those accessory proteins for which it has been identified upstream of the start codon is also mentioned.

ORF	Coordinates	Gene Length (nt)	CDS Length (nt)	TRS Location
3a	25,393–26,220	828	275	25,379
3b	25,765–26,220	456	151	
6	27,202–27,387	186	61	27,035
7a	27,394–27,759	366	121	27,382
7b	27,756–27,887	132	43	
8	27,894–28,259	366	121	27,882
9a	28,284–28,577	294	97	
9b	28,734–28,955	222	73	
10	29,558–29,764	117	38	29,528

**Table 2:** High sequence identity homologs of ORF8 protein.

Protein	Accession	Length [Coverage]	Percent Identity	Host	Isolate	Genome Accession	Genome Identity
Non-structural [NS8]	QHR63307.1	121[100]	95.04	Bat	CoV-Ra TG13	MN996532	95.98
Hypothetical	AVP78037.1	121[100]	94.21	Bat	SL-CoVZC45	MG772933	84.69
ORF8	QIA48620.1	121[100]	87.60	Pangolin	PCoV-GX-P4L	MT040333.1	80.17
ORF8	QIG55952.1	105[100]	81.82	Pangolin	CoV-Isolate MP789	MT121216.1	86.66

**Table 3:** Genome identities among human, bat, and pangolin coronavirus isolates in which SARS-CoV-2 ORF8 high identity homologs are detected.

Subject	Target	Genome Identity
**SARS-CoV-2**	Bat-CoV-Ra TG13	95.98
	Bat-SL-CoVZC45	84.69
	Pangolin-CoV-GX-789	86.66
	Pangolin-CoV-GX-P4L	80.17
**Bat-CoV-Ra TG13**	Bat-SL-CoVZC45 Pangolin-CoV-MP789	85.3 89.3
	Pangolin-CoV-GX-P4L	79.9

**Table 4:** Recombination regions identified from RDP4 analysis in which SARS-CoV-2 is the recombinant sequence.

No.	Begin	End	Recombinant Sequence(s)	Minor Parental Sequence(s)	Major Parental Sequence(s)	RDP *p*-Value
1	927	1708	SARS-CoV-2	Bat-SL-CoVZC45	Pangolin-CoV-MP789	2.00 × 10^−6^
2	1935	3194	SARS-CoV-2	Bat-SL-CoVZC45	Pangolin-CoV-MP789	3.05 × 10^−11^
3	3664	4363	SARS-CoV-2	Bat-SL-CoVZC45	Pangolin-CoV-MP789	3.84 × 10^−9^
4	22,874	23,092	SARS-CoV-2	Pangolin-CoV-GX-P4L	Bat-SL-CoVZC45	2.52 × 10^−2^
5	23,156	23,306	SARS-CoV-2	Pangolin-CoV-GX-P4L	Bat-SL-CoVZC45	5.09 × 10^−3^
6	23,898	24,248	Bat-CoV-RaTG13	Pangolin-CoV-GX-P4L	Bat-SL-CoVZC45	1.47 × 10^−3^
			SARS-CoV-2			
7	6649	6833	Bat-CoV-RaTG13	Bat-SL-CoVZC45	Pangolin-CoV-MP789	3.19 × 10^−2^
			SARS-CoV-2			

**Table 5:** Recombination regions identified from RDP4 analysis in which SARS-CoV-2 is involved either as major parent or minor parent.

Number	Begin	End	Recombinant Sequence(s)	Minor Parental Sequence(s)	Major Parental Sequence(s)	RDP *p*-Value
1	380	11,623	Pangolin-CoV-GX-P4L	Unknown (Bat-SL-CoVZC45)	SARS-CoV-2	1.27 × 10^−57^
2	7054	8258	Pangolin-CoV-MP789	Unknown (Bat-SL-CoVZC45)	SARS-CoV-2	1.36 × 10^−10^
3	9558	9947	Pangolin-CoV-MP789	Bat-SL-CoVZC45	SARS-CoV-2	2.20 × 10^−2^
4	14,611	15,451	Pangolin-CoV-MP789	Unknown (Bat-SL-CoVZC45)	SARS-CoV-2	8.00 × 10^−13^
5	17,813	18,698	Bat-SL-CoVZC45	Unknown (Pangolin-CoV-GX-P4L)	SARS-CoV-2	9.99 × 10^−3^
6	19,847	19,963	Pangolin-CoV-MP789	Pangolin-CoV-GX-P4L	SARS-CoV-2	5.09 × 10^−9^
7	21,563	21,904	Pangolin-CoV-MP789	Unknown (Pangolin-CoV-GX-P4L)	SARS-CoV-2	1.34 × 10^−9^
8	21,914	22,474	Pangolin-CoV-MP789	Unknown (Pangolin-CoV-GX-P4L)	SARS-CoV-2	3.73 × 10^−21^
9	22,850	23,094	Bat-CoV-RaTG13	Unknown (Pangolin-CoV-MP789)	SARS-CoV-2	1.70 × 10^−16^
10	4816	5953	Pangolin-CoV-MP789	Unknown (Bat-SL-CoVZC45)	Bat-CoV-RaTG13	1.25 × 10^−9^
					SARS-CoV-2	
11	14,042	14,607	Bat-SL-CoVZC45	Unknown (Pangolin-CoV-GX-P4L)	Pangolin-CoV-MP789	2.13 × 10^−4^
					SARS-CoV-2	
					Bat-CoV-RaTG13	
12	16,028	16,399	Bat-SL-CoVZC45	Unknown (Pangolin-CoV-GX-P4L)	Bat-CoV-RaTG13	2.07 × 10^−4^
					SARS-CoV-2	
13	21,187	22,368	Bat-SL-CoVZC45	Pangolin-CoV-MP789	Bat-CoV-RaTG13	1.69 × 10^−45^
					SARS-CoV-2	
14	20,015	20,591	Bat-SL-CoVZC45	Unknown (Pangolin-CoV-GX-P4L)	Bat-CoV-RaTG13	1.42 × 10^−7^
					SARS-CoV-2	
15	22,472	22,792	Pangolin-CoV-GX-P4L	Bat-CoV-RaTG13	Unknown (Bat-SL-CoVZC45)	3.91 × 10^−4^
				SARS-CoV-2		

**Table 6:** Summary of SARS-CoV-2 ORF8 and SARS-CoV-ORF8ab features. Pictorial depiction of biochemical/structural features can be viewed in Figure 5.

Characteristic	SARS-CoV ORF8ab	SARS-COV-2 ORF8
Nucleotide Identity	26%	
Protein Identity	20%	
Nucleotide Deletion	Yes	No (This Study)
Origin	Bat	Bat, Pangolin (This Study)
Biochemical/Structural Features		
N-Terminal Peptide Sequence	Yes	Yes (Predicted in this Study)
N-Glycosylation Site	Yes	Yes (Identified in this Study)
Cysteine Residues	Yes	Yes
Localization	Endoplasmic Reticulum	Endoplasmic Reticulum (Predicted/proposed in this Study)
Protein Family Conserved Motifs	Yes	Yes
Macromolecular Interactions	Protein–protein	Protein–protein and Protein–DNA
Functional Features		
Viral Replication	Yes	Not studied so far
Host Immune Modulation	Yes	Yes
Protein Quality Control	Yes	Yes

## Supplementary Materials

The following are available online at https://www.mdpi.com/2076-0817/9/9/677/s1, Figure S1: 29-nt deletion in SARS-CoV ORF8ab, Figure S2: SVG File for Multiple Sequence Alignment of NCBI Sequence Dataset, Figure S3: SVG File for Multiple Sequence Alignment of GISAID Sequence Dataset, Table S1: NCBI Dataset Information, Table S2: GISAID Dataset Information, Material S1: Supplementary Methods.
