# Supplementary material for: SARS-CoV-2 ORF8 and SARS-CoV ORF8ab: Genomic Divergence and Functional Convergence
Source: Pathogens. 2020 Aug 20;9(9):677. doi: 10.3390/pathogens9090677 (PMC7558349; doi:10.3390/pathogens9090677)
Supplement: Supplementary file 1 [file pathogens-09-00677-s001.zip › Supplementary Files/Figure S1. 29-nt deletion in SARS-CoV ORF8ab.pdf]

```
             1        10        20        30        40        50
AY278489     ATGAAACTTCTCATTGTTTTGACTTGTATTTCTCTATGCAGTTGCATACG
AY283798     ATGAAACTTCTCATTGTTTTGACTTGTATTTCTCTATGCAGTTGCATATG

                      60        70        80        90       100
AY278489     CACTGTAGTACAGCGCTGTGCATCTAATAAACCTCATGTGCTTGAAGATC
AY283798     CACTGTAGTACAGCGCTGTGCATCTAATAAACCTCATGTGCTTGAAGATC

                     110       120       130       140       150
AY278489     CTTGTCCTACTGGTTACCAACCTGAATGGAATATAAGGTACAACACTAGG
AY283798     CTTGT.............................AAGGTACAACACTAGG

                     160       170       180       190       200
AY278489     GGTAATACTTATAGCACTGCTTGGCTTTGTGCTCTAGGAAAGGTTTTACC
AY283798     GGTAATACTTATAGCACTGCTTGGCTTTGTGCTCTAGGAAAGGTTTTACC

                     210       220       230       240       250
AY278489     TTTTCATAGATGGCACACTATGGTTCAAACATGCACACCTAATGTTACTA
AY283798     TTTTCATAGATGGCACACTATGGTTCAAACATGCACACCTAATGTTACTA

                     260       270       280       290       300
AY278489     TCAACTGTCAAGATCCAGCTGGTGGTGCGCTTATAGCTAGGTGTTGGTAC
AY283798     TCAACTGTCAAGATCCAGCTGGTGGTGCGCTTATAGCTAGGTGTTGGTAC

                     310       320       330       340       350
AY278489     CTTCATGAAGGTCACCAAACTGCTGCATTTAGAGACGTATTTGTTGTTTT
AY283798     CTTCATGAAGGTCACCAAACTGCTGCATTTAGAGACGTACTTGTTGTTTT

                     360
AY278489     AAATAAACGAACAAATTAA
AY283798     AAATAAACGAACAAATTAA
```
