# Supplementary material for: SARS-CoV-2 ORF8 and SARS-CoV ORF8ab: Genomic Divergence and Functional Convergence
Source: Pathogens. 2020 Aug 20;9(9):677. doi: 10.3390/pathogens9090677 (PMC7558349; doi:10.3390/pathogens9090677)
Supplement: Supplementary file 1 [file pathogens-09-00677-s001.zip › Supplementary Files/Table S1. NCBI DATASET INFORMATION.pdf]

S2. Details of the sequences in NCBI Dataset. Information has been retrieved from https://www.ncbi.nlm.nih.gov/labs/virus/vssi/#/virus?SeqType_s=Nucleotide&VirusLineage_ss=Wuhan%20seafood%20market%20pneumonia%20virus,%20taxid:2697049

Accession	Geo_Location	Collection_Date
MN908947	China	2019-12
NC_045512	China	2019-12
MT291829	China: Wuhan	12/30/2019
MT186683	Hong Kong	2020-01
MT270104	Germany: Bavaria	2020-01
MT019533	China: Hubei, Wuhan	1/1/2020
MN988669	China	1/2/2020
MT093631	China	1/8/2020
MN938384	China: Shenzhen	1/10/2020
MT072688	Nepal	1/13/2020
MT192773	Viet Nam: Ho Chi Minh city	1/22/2020
MT039874	China	1/22/2020
MT007544	Australia: Melbourne, Victoria	1/25/2020
MT020880	USA: WA	1/25/2020
MT192759	Taiwan	1/25/2020
MT407653	China: Zhejiang	1/26/2020
MT044258	USA: CA	1/27/2020
MT012098	India: Kerala State	1/27/2020
MT114415	Hong Kong	1/27/2020
MT135042	China: Beijing	1/28/2020
MT020781	Finland	1/29/2020
MT039888	USA: MA	1/29/2020
MT066156	Italy	1/30/2020
MT066175	Taiwan	1/31/2020
MT077125	Italy	1/31/2020
MT121215	China: Shanghai	2/2/2020
MT374101	Taiwan	2/4/2020
MT446312	China: Guangdong, Guangzhou	2/5/2020
MT093571	Sweden	2/7/2020
MT106054	USA: TX	2/11/2020
MT152824	USA: Snohomish County, WA	2/24/2020
MT159716	USA	2/24/2020
MT412134	China	2/24/2020
MT233519	Spain: Valencia	2/27/2020
MT304474	South Korea	2/27/2020
MT370518	Taiwan	2/27/2020
MT126808	Brazil	2/28/2020

MT582499	Germany: Heinsberg	2/28/2020
MT304475	South Korea	2/29/2020
MT320538	France	2020-03
MT322394	USA: VA	2020-03
MT163720	USA: WA	3/1/2020
MT325565	USA: FL	3/2/2020
MT240479	Pakistan: Gilgit	3/4/2020
MT370842	USA: NY	3/4/2020
MT188339	USA: MN	3/9/2020
MT292571	Spain	3/9/2020
MT320891	Iran	3/9/2020
MT510999	Netherlands: Leiden	3/9/2020
MT371048	Sri Lanka	3/10/2020
MT252680	USA: WA	3/10/2020
MT252683	USA: WA	3/10/2020
MT512416	USA	3/10/2020
MT678839	South Korea	3/11/2020
MT246451	USA: WA	3/13/2020
MT246461	USA: WA	3/13/2020
MT246482	USA: WA	3/15/2020
MT359865	Spain	3/15/2020
MT507281	USA	3/15/2020
MT510718	USA	3/15/2020
MT500122	Pakistan: Karachi	3/16/2020
MT507794	Jamaica	3/16/2020
MT670018	Chile	3/17/2020
MT679198	USA: NY	3/17/2020
MT350282	Brazil	3/18/2020
MT371049	Sri Lanka	3/19/2020
MT371569	Czech Republic	3/19/2020
MT372482	Malaysia	3/20/2020
MT479226	Taiwan	3/20/2020
MT371570	Czech Republic	3/21/2020
MT520360	USA: Massachusetts	3/22/2020
MT635445	Russia: Moscow region	3/22/2020
MT637143	Russia: Moscow region	3/22/2020
MT451195	Australia: Victoria	3/23/2020
MT451207	Australia: Victoria	3/24/2020
MT559038	Tunisia: Ben Arous	3/24/2020
MT375429	USA: ID	3/29/2020
MT293156	USA: WA	3/30/2020
MT415904	USA: VA	2020-04
MT511067	Poland	4/2/2020

MT461646	USA: WA	4/3/2020
MT358637	India: Rajkot	4/5/2020
MT358401	USA: New Orleans, LA	4/6/2020
MT641731	Australia: Victoria	4/8/2020
MT675958	Turkey	4/8/2020
MT499210	Poland	4/11/2020
MT435079	India: Ahmedabad	4/13/2020
MT641759	Australia: Victoria	4/14/2020
MT641776	Timor-Leste	4/14/2020
MT605818	Turkey	4/16/2020
MT435086	India: Mansa	4/21/2020
MT435085	India: Gandhinagar	4/22/2020
MT438739	USA: CA	4/22/2020
MT396266	Netherlands: Milheeze	4/24/2020
MT451881	India: Ahmedabad	4/26/2020
MT577627	USA: FL	4/28/2020
MT510690	Egypt	5/2/2020
MT496992	India: Ahmedabad	5/3/2020
MT502774	Bangladesh	5/13/2020
MT539159	Bangladesh	5/21/2020
MT601286	Bangladesh: Dhaka	6/1/2020
MT614597	Egypt	6/2/2020
MT635672	Bangladesh	6/7/2020
MT039890	South Korea	2020-01
