# Supplementary material for: SARS-CoV-2 ORF8 and SARS-CoV ORF8ab: Genomic Divergence and Functional Convergence
Source: Pathogens. 2020 Aug 20;9(9):677. doi: 10.3390/pathogens9090677 (PMC7558349; doi:10.3390/pathogens9090677)
Supplement: Supplementary file 1 [file pathogens-09-00677-s001.zip › Supplementary Files/Table S2. GISAID DATASET INFORMATION.pdf]

S3. Details of the sequences in GISAID Dataset. Information has been retrieved from https://www.epicov.org/epi3/frontend#48b3ad

Accession ID	Virus name	Location	Collection date
EPI_ISL_402123	hCoV-19/Wuhan/IPBCAMS-WH-01/2019	Asia / China / Hubei / Wuhan	2019-12-24
EPI_ISL_402127	hCoV-19/Wuhan/WIV02/2019	Asia / China / Hubei / Wuhan	2019-12-30
EPI_ISL_402129	hCoV-19/Wuhan/WIV06/2019	Asia / China / Hubei / Wuhan	2019-12-30
EPI_ISL_403932	hCoV-19/Guangdong/20SF012/2020	Asia / China / Guandong / Shenzhen	2020-01-14
EPI_ISL_406534	hCoV-19/Foshan/20SF207/2020	Asia / China / Guangdong / Foshan	2020-01-22
EPI_ISL_428469	hCoV-19/Guangdong/20SF665/2020	Asia / China / Guangdong / Zhongshan	2020-01-27
EPI_ISL_428444	hCoV-19/Guangdong/20SF1152/2020	Asia / China / Guangdong / Huizhou	2020-01-30
EPI_ISL_416342	hCoV-19/Shanghai/SH0033/2020	Asia / China / Shanghai	2020-02-04
EPI_ISL_431782	hCoV-19/Fujian/IM3520014T/2020	Asia / China / Fujian	2020-03-22
EPI_ISL_406973	hCoV-19/Singapore/1/2020	Asia / Singapore	2020-01-23
EPI_ISL_418996	hCoV-19/Singapore/15/2020	Asia / Singapore	2020-01-27
EPI_ISL_419001	hCoV-19/Singapore/19/2020	Asia / Singapore	2020-03-02
EPI_ISL_435695	hCoV-19/Singapore/90/2020	Asia / Singapore	2020-04-23
EPI_ISL_420555	hCoV-19/India/c32/2020	Asia / India	2020-03-03
EPI_ISL_435106	hCoV-19/India/NCDC-01760/2020	Asia / India / Ladakh	2020-03-18
EPI_ISL_436453	hCoV-19/India/NCDC-4444/2020	Asia / India / Madhya Pradesh	2020-04-16
EPI_ISL_437442	hCoV-19/India/GBRC14/2020	Asia / India / Gujarat / Surat	2020-04-27
EPI_ISL_416432	hCoV-19/Saudi Arabia/KAIMRC-Alghoribi/2020	Asia / Saudi Arabia / Riyadh	2020-03-07
EPI_ISL_416521	hCoV-19/Saudi Arabia/SCDC-3321/2020	Asia / Saudi Arabia	2020-03-10
EPI_ISL_437459	hCoV-19/Saudi Arabia/KAUST-MAKKAH03/2020	Asia / Saudi Arabia / Makkah	2020-03-23
EPI_ISL_437469	hCoV-19/Saudi Arabia/KAUST-MADINAH25/2020	Asia / Saudi Arabia / Madinah	2020-03-29
EPI_ISL_437475	hCoV-19/Saudi Arabia/KAUST-JEDDAH64/2020	Asia / Saudi Arabia / Jeddah	2020-04-01
EPI_ISL_435281	hCoV-19/Indonesia/JKT-EIJK0141/2020	Asia / Indonesia / Jakarta	2020-03-17
EPI_ISL_437188	hCoV-19/Indonesia/EJ-ITD3590NT/2020	Asia / Indonesia / Surabaya	2020-04-14
EPI_ISL_417920	hCoV-19/Malaysia/190300/2020	Asia / Malaysia / Kuala Lumpur	2020-03-22
EPI_ISL_430439	hCoV-19/Malaysia/IMR_WC1177/2020	Asia / Malaysia	2020-03-05
EPI_ISL_413515	hCoV-19/South Korea/KUMC05/2020	Asia / South Korea	2020-02-27
EPI_ISL_425118	hCoV-19/Korea/KCDC2002/2020	Asia / South Korea	2020-01-30
EPI_ISL_427812	hCoV-19/Korea/KCDC2018/2020	Asia / South Korea	2020-02-19
EPI_ISL_404895	hCoV-19/USA/WA1/2020	North America / USA / Washington / Snohomish County	2020-01-19
EPI_ISL_413014	hCoV-19/Canada/ON-PHL2445/2020	North America / Canada / Ontario	2020-01-25
EPI_ISL_410044	hCoV-19/USA/CA6/2020	North America / USA / California	2020-01-27
EPI_ISL_410045	hCoV-19/USA/IL2/2020	North America / USA / Illinois	2020-01-28

EPI_ISL_409067	hCoV-19/USA/MA1/2020	North America / USA / Massachusetts	2020-01-29
EPI_ISL_413613	hCoV-19/USA/CruiseA-8/2020	North America / USA	2020-02-17
EPI_ISL_412972	hCoV-19/Mexico/CDMX-InDRE_01/2020	North America / Mexico / Mexico City	2020-02-27
EPI_ISL_415581	hCoV-19/Canada/BC_02421/2020	North America / Canada / British Columbia	2020-03-01
EPI_ISL_420797	hCoV-19/USA/TX_2817/2020	North America / USA / Texas	2020-03-01
EPI_ISL_436050	hCoV-19/USA/NY-SURV0144/2020	North America / USA / New York / New York City	2020-03-02
EPI_ISL_418330	hCoV-19/Canada/ON_PHL8580/2020	North America / Canada / Ontario	2020-03-05
EPI_ISL_419261	hCoV-19/USA/VA-DCLS-0009/2020	North America / USA / Virginia	2020-03-09
EPI_ISL_424868	hCoV-19/USA/LA_0842/2020	North America / USA / Louisiana	2020-03-09
EPI_ISL_436052	hCoV-19/USA/NY-SURV0165/2020	North America / USA / New York / New York City	2020-03-09
EPI_ISL_414648	hCoV-19/USA/CA-PC101P/2020	North America / USA / California / San Diego County	2020-03-11
EPI_ISL_417192	hCoV-19/USA/MN25-MDH25/2020	North America / USA / Minnesota	2020-03-12
EPI_ISL_426365	hCoV-19/Mexico/CDMX-INCMNSZ_05/2020	North America / Mexico / Mexico City	2020-03-12
EPI_ISL_427530	hCoV-19/USA/NY-WCMP3E02P/2020	North America / USA / New York / Manhattan	2020-03-12
EPI_ISL_417027	hCoV-19/USA/UT-09/2020	North America / USA / Utah	2020-03-20
EPI_ISL_418350	hCoV-19/Canada/ON_PHL3380/2020	North America / Canada / Ontario	2020-03-20
EPI_ISL_428316	hCoV-19/USA/WI-220/2020	North America / USA / Wisconsin / Mequon	2020-03-25
EPI_ISL_427162	hCoV-19/USA/CT-UW-4238/2020	North America / USA / Connecticut	2020-03-30
EPI_ISL_428757	hCoV-19/USA/NY-NYUMC243/2020	North America / USA / New York / Brooklyn	2020-03-30
EPI_ISL_430940	hCoV-19/USA/ID-UW-4255/2020	North America / USA / Idaho	2020-03-30
EPI_ISL_435444	hCoV-19/USA/AK-PHL193/2020	North America / USA / Alaska / Wasilla	2020-04-09
EPI_ISL_435517	hCoV-19/USA/NY-NYUMC450/2020	North America / USA / New York / Nassau County	2020-04-20
EPI_ISL_435474	hCoV-19/USA/CA-SR0176/2020	North America / USA / California / San Diego	2020-04-29
EPI_ISL_412116	hCoV-19/England/09c/2020	Europe / United Kingdom / England	2020-02-09
EPI_ISL_412973	hCoV-19/Italy/CDG1/2020	Europe / Italy / Lombardy	2020-02-20
EPI_ISL_413996	hCoV-19/Switzerland/TI9486/2020	Europe / Switzerland / Tessin	2020-02-24
EPI_ISL_414497	hCoV-19/Germany/NRW-02-1/2020	Europe / Germany / North Rhine Westphalia / Heinsberg District	2020-02-25
EPI_ISL_414623	hCoV-19/France/GE1583/2020	Europe / France / Grand-Est / Strasbourg	2020-02-25
EPI_ISL_418251	hCoV-19/Spain/Madrid201105/2020	Europe / Spain / Madrid	2020-02-25
EPI_ISL_416142	hCoV-19/Denmark/SSI-01/2020	Europe / Denmark / Copenhagen	2020-02-26
EPI_ISL_417484	hCoV-19/Norway/1380/2020	Europe / Norway / Oslo	2020-02-26
EPI_ISL_419655	hCoV-19/Austria/CeMM0002/2020	Europe / Austria / Vienna	2020-02-26
EPI_ISL_413555	hCoV-19/Wales/PHW1/2020	Europe / United Kingdom / Wales	2020-02-27
EPI_ISL_425551	hCoV-19/England/NOTT-10E49C/2020	Europe / United Kingdom / England	2020-03-22

EPI_ISL_432348	hCoV-19/Wales/PHWC-25339/2020	Europe / United Kingdom / Wales	2020-03-23
EPI_ISL_425893	hCoV-19/Scotland/EDB103/2020	Europe / United Kingdom / Scotland	2020-03-22
EPI_ISL_433866	hCoV-19/England/CAMB-7ACFE/2020	Europe / United Kingdom / England	2020-04-08
EPI_ISL_433426	hCoV-19/Scotland/EDB1182/2020	Europe / United Kingdom / Scotland	2020-04-08
EPI_ISL_433481	hCoV-19/England/CAMB-7FB56/2020	Europe / United Kingdom / England	2020-04-17
EPI_ISL_417922	hCoV-19/Italy/INMI4/2020	Europe / Italy / Rome	2020-02-28
EPI_ISL_418256	hCoV-19/Italy/TE4880/2020	Europe / Italy / Abruzzo	2020-03-14
EPI_ISL_435147	hCoV-19/Italy/TE12759/2020	Europe / Italy / Abruzzo	2020-04-08
EPI_ISL_406862	hCoV-19/Germany/BavPat1/2020	Europe / Germany / Bavaria / Munich	2020-01-28
EPI_ISL_419534	hCoV-19/Germany/NRW-17/2020	Europe / Germany / Duesseldorf	2020-03-11
EPI_ISL_437237	hCoV-19/Germany/BAV-MVP0038/2020	Europe / Germany / Bavaria / Munich	2020-03-23
EPI_ISL_437297	hCoV-19/Germany/BAV-MVP0107/2020	Europe / Germany / Bavaria / Munich	2020-04-13
EPI_ISL_406596	hCoV-19/France/IDF0372/2020	Europe / France / Ile-de-France / Paris	2020-01-23
EPI_ISL_429968	hCoV-19/France/HF1463/2020	Europe / France / Hauts de France / Compiegne	2020-02-21
EPI_ISL_416495	hCoV-19/France/HF2234/2020	Europe / France / Hauts de France / Compiègne	2020-03-10
EPI_ISL_419170	hCoV-19/France/ARA11943/2020	Europe / France / ARA / Lyon	2020-03-21
EPI_ISL_428366	hCoV-19/France/HF4220/2020	Europe / France / Hauts De France / Chateau-thierry	2020-03-30
EPI_ISL_434633	hCoV-19/France/OCC-18/2020	Europe / France / Occitanie	2020-04-09
EPI_ISL_419677	hCoV-19/Spain/Valencia13/2020	Europe / Spain / Comunitat Valenciana / Valencia	2020-03-09
EPI_ISL_425218	hCoV-19/Spain/Valencia94/2020	Europe / Spain / Comunitat Valenciana / Valencia	2020-03-17
EPI_ISL_428700	hCoV-19/Spain/Madrid_H12_2501/2020	Europe / Spain / Madrid	2020-03-27
EPI_ISL_419562	hCoV-19/Luxembourg/LNS0000001/2020	Europe / Luxembourg	2020-02-29
EPI_ISL_430469	hCoV-19/Greece/127_HPI/2020	Europe / Greece / Athens	2020-02-29
EPI_ISL_413588	hCoV-19/Netherlands/Utrecht_1363564/2020	Europe / Netherlands / Utrecht	2020-03-01
EPI_ISL_415154	hCoV-19/Belgium/BM-03012/2020	Europe / Belgium / Kraainem	2020-03-01
EPI_ISL_417736	hCoV-19/Iceland/14/2020	Europe / Iceland / Reykjavik	2020-03-01
EPI_ISL_421487	hCoV-19/Portugal/PT0084/2020	Europe / Portugal	2020-03-22
EPI_ISL_434670	hCoV-19/Sweden/20-07439/2020	Europe / Sweden / Vastra Gotaland	2020-04-17
EPI_ISL_416741	hCoV-19/Lithuania/ChVir1632/2020	Europe / Lithuania / Vilnius	2020-02
EPI_ISL_415710	hCoV-19/Russia/StPetersburg-3524/2020	Europe / Russia / Saint Petersburg	2020-03-15
EPI_ISL_420080	hCoV-19/Russia/StPetersburg-RII3992/2020	Europe / Russia / Saint Petersburg	2020-03-18
EPI_ISL_427310	hCoV-19/Russia/StPetersburg-RII4382V/2020	Europe / Russia / Saint-Petersburg	2020-03-29
EPI_ISL_430086	hCoV-19/Russia/StPetersburg-RII5033S/2020	Europe / Russia / St.Petersburg	2020-04-10

EPI_ISL_430110	hCoV-19/Russia/StPetersburg-RII6065S/2020	Europe / Russia / St.Petersburg	2020-04-15
EPI_ISL_430112	hCoV-19/Russia/Ulan-Ude-RII4562V/2020	Europe / Russia / Buryat Republic / Ulan-Ude	2020-03-25
EPI_ISL_408976	hCoV-19/Australia/NSW02/2020	Oceania / Australia / New South Wales / Sydney	2020-01-22
EPI_ISL_416519	hCoV-19/New Zealand/20VR0189/2020	Oceania / New Zealand / Auckland	2020-03-02
EPI_ISL_416539	hCoV-19/New Zealand/20VR0276/2020	Oceania / New Zealand / Wellington	2020-03-15
EPI_ISL_419796	hCoV-19/Australia/VIC86/2020	Oceania / Australia / Victoria	2020-03-15
EPI_ISL_412964	hCoV-19/Brazil/SPBR-01/2020	South America / Brazil / Sao Paulo / Sao Paulo	2020-02-25
EPI_ISL_415661	hCoV-19/Chile/Santiago_op4d1/2020	South America / Chile / Santiago	2020-03-08
EPI_ISL_415787	hCoV-19/Peru/010/2020	South America / Peru / Lima	2020-03-10
EPI_ISL_417924	hCoV-19/Colombia/Antioquia79256/2020	South America / Colombia / Antioquia	2020-03-11
EPI_ISL_430816	hCoV-19/Argentina/PAIS_A025/2020	South America / Argentina / Ciudad Autonoma de Buenos Aires	2020-04-18
EPI_ISL_418206	hCoV-19/Senegal/003/2020	Africa / Senegal / Dakar	2020-02-28
EPI_ISL_418241	hCoV-19/Algeria/G0638_2264/2020	Africa / Algeria / Boufarik	2020-03-02
EPI_ISL_420852	hCoV-19/DRC/396/2020	Africa / Democratic Republic of the Congo / Kinshasa	2020-03-26
EPI_ISL_421573	hCoV-19/South Africa/KRISP-06/2020	Africa / South Africa / KZN	2020-03-31
EPI_ISL_428857	hCoV-19/Gambia/GC19-029/2020	Africa / Gambia / West Coast Region	2020-04-20
EPI_ISL_430297	hCoV-19/South Africa/R02827/2020	Africa / South Africa / GP	2020-03-06
EPI_ISL_456713	hCoV-19/England/CAMB-1B2C08/2020	Europe / United Kingdom / England	2020-05-19
EPI_ISL_457482	hCoV-19/England/NORW-EBB64/2020	Europe / United Kingdom / England	2020-05-06
EPI_ISL_458150	hCoV-19/Morocco/15N/2020	Africa / North Africa / Morocco	2020-05-15
EPI_ISL_459893	hCoV-19/Luxembourg/LNS0681882/2020	Europe / Luxembourg	2020-05-15
EPI_ISL_460888	hCoV-19/Netherlands/Gelderland_127/2020	Europe / Netherlands / Gelderland	2020-05-22
EPI_ISL_461759	hCoV-19/Scotland/EDB5590/2020	Europe / United Kingdom / Scotland	2020-05-25
EPI_ISL_463694	hCoV-19/USA/WA-S1204/2020	North America / USA / Washington	2020-05-06
EPI_ISL_463696	hCoV-19/USA/WA-S1206/2020	North America / USA / Washington	2020-05-06
EPI_ISL_466626	hCoV-19/Bangladesh/BCSIR-NILMRC_051/2020	Asia / Bangladesh	2020-05-07
EPI_ISL_466909	hCoV-19/Germany/BAV-MVP0283/2020	Europe / Germany / Bavaria / Munich	2020-05-11
EPI_ISL_467190	hCoV-19/Spain/COV003722/2020	Europe / Spain	2020-05-07
EPI_ISL_468465	hCoV-19/USA/CA-CZB-1519/2020	North America / USA / California	2020-05-11
EPI_ISL_469055	hCoV-19/Sweden/20-14262/2020	Europe / Sweden / Trangsund / Spelvagen	2020-05-02
EPI_ISL_469275	hCoV-19/Egypt/CUNCI-HGC002-2/2020	North Africa / Egypt	2020-05-02
EPI_ISL_471702	hCoV-19/USA/MI-MDHHS-SC20678/2020	North America / USA / Michigan	2020-05-14

EPI_ISL_473127	hCoV-19/Wales/PHWC-162830/2020	Europe / United Kingdom / Wales	2020-05-31
EPI_ISL_473676	hCoV-19/Scotland/CVR3539/2020	Europe / United Kingdom / Scotland	2020-05-13
EPI_ISL_473926	hCoV-19/Scotland/EDB6244/2020	Europe / United Kingdom / Scotland	2020-05-21
EPI_ISL_475754	hCoV-19/Bangladesh/BCSIR-NILMRC_025/2020	Asia / Bangladesh	2020-05-24
EPI_ISL_477001	hCoV-19/Belgium/rega-0505428/2020	Europe / Belgium / Rega	2020-05-05
EPI_ISL_477004	hCoV-19/Belgium/rega-0506487/2020	Europe / Belgium / Rega	2020-05-06
EPI_ISL_477169	hCoV-19/Georgia/Tb-7851/2020	Europe / Georgia / Tbilisi	2020-05-01
EPI_ISL_478094	hCoV-19/Scotland/CVR3690/2020	Europe / United Kingdom / Scotland	2020-05-24
EPI_ISL_479519	hCoV-19/India/NIV-22552/2020	Asia / India	2020-05-07
EPI_ISL_480274	hCoV-19/Turkey/GLAB-CoV187/2020	Europe / Turkey / Istanbul	2020-05-07
EPI_ISL_480305	hCoV-19/Bulgaria/27/2020	Europe / Bulgaria	2020-05-17
EPI_ISL_480413	hCoV-19/USA/WI-UW-431/2020	North America / USA / Wisconsin	2020-05-01
EPI_ISL_481907	hCoV-19/England/BRIS-12D206/2020	Europe / United Kingdom / England	2020-05-01
EPI_ISL_482501	hCoV-19/India/NCDC1722_CSIR-IGIB/2020	Asia / India	2020-05-17
EPI_ISL_482669	hCoV-19/India/NCDC7757_CSIR-IGIB/2020	Asia / India	2020-05-28
EPI_ISL_483325	hCoV-19/USA/CA-ALSR-0724-IPL/2020	North America / USA / California	2020-05-04
EPI_ISL_467053	hCoV-19/India/GBRC183a/2020	Asia / India	2020-06-03
EPI_ISL_469048	hCoV-19/India/GBRC202/2020	Asia / India	2020-06-05
EPI_ISL_472090	hCoV-19/England/LIVE-A00F3/2020	Europe / United Kingdom / England	2020-06-05
EPI_ISL_472354	hCoV-19/England/NORW-ED18E/2020	Europe / United Kingdom / England	2020-06-01
EPI_ISL_477762	hCoV-19/England/BIRM-61331/2020	Europe / United Kingdom / England	2020-06-13
EPI_ISL_478392	hCoV-19/England/LIVE-A0C2C/2020	Europe / United Kingdom / England	2020-06-15
EPI_ISL_479242	hCoV-19/England/SHEF-CA71C/2020	Europe / United Kingdom / England	2020-06-03
EPI_ISL_479246	hCoV-19/England/SHEF-CB168/2020	Europe / United Kingdom / England	2020-06-16
EPI_ISL_479560	hCoV-19/India/NIV-64908/2020	Asia / India	2020-06-16
EPI_ISL_480676	hCoV-19/Australia/VIC1952/2020	Oceania / Australia / Victoria	2020-06-09
EPI_ISL_480704	hCoV-19/Australia/VIC2093/2020	Oceania / Australia / Victoria	2020-06-15
EPI_ISL_481183	hCoV-19/India/ILSCV34479/2020	Asia / India	2020-06-09
EPI_ISL_481242	hCoV-19/USA/MN-MDH-1224/2020	North America / USA / Minnesota	2020-06-06
EPI_ISL_482699	hCoV-19/Singapore/552/2020	Asia / Singapore	2020-06-06
EPI_ISL_482867	hCoV-19/SouthAfrica/KRISP-0280/2020	Africa / South Africa / KZN	2020-06-16
EPI_ISL_483504	hCoV-19/USA/CA-ALSR-1077-SAN/2020	North America / USA / California	2020-06-22
EPI_ISL_483619	hCoV-19/Singapore/596/2020	Asia / Singapore	2020-06-22
EPI_ISL_483687	hCoV-19/Bangladesh/BCSIR-NILMRC_205/2020	Asia / Bangladesh	2020-06-26
EPI_ISL_484672	hCoV-19/Scotland/CVR3886/2020	Europe / United Kingdom / Scotland	2020-06-03
EPI_ISL_484684	hCoV-19/Scotland/EDB6609/2020	Europe / United Kingdom / Scotland	2020-06-22
EPI_ISL_484689	hCoV-19/Wales/PHWC-165615/2020	Europe / United Kingdom / Wales	2020-06-28
EPI_ISL_484939	hCoV-19/USA/WI-UW-564/2020	North America / USA / Wisconsin	2020-06-28
EPI_ISL_484974	hCoV-19/USA/WI-UW-599/2020	North America / USA / Wisconsin	2020-06-28

EPI_ISL_486117	hCoV-19/USA/CA-CZB-1783/2020	North America / USA / California	2020-06-03
EPI_ISL_486514	hCoV-19/Switzerland/160021_450_B01/2020	Europe / Switzerland	2020-06-15
EPI_ISL_486516	hCoV-19/Switzerland/170001_454_D09/2020	Europe / Switzerland	2020-06-27
EPI_ISL_486850	hCoV-19/Ecuador/USFQ-118/2020	South America / Ecuador / San Francisco	2020-06-30
EPI_ISL_487106	hCoV-19/Nigeria/ON191-CV49/2020	Africa / Nigeria	2020-06-30
EPI_ISL_487273	hCoV-19/Bahrain/110003611/2020	Asia / Bahrain	2020-06-25
