# Supplementary material for: SARS-CoV-2 ORF8 and SARS-CoV ORF8ab: Genomic Divergence and Functional Convergence
Source: Pathogens. 2020 Aug 20;9(9):677. doi: 10.3390/pathogens9090677 (PMC7558349; doi:10.3390/pathogens9090677)
Supplement: Supplementary file 1 [file pathogens-09-00677-s001.zip › Supplementary Files/Text S1. SUPPLEMENTARY Method and References.pdf]

Methods: Structural analysis was performed using the PSI-blast-based secondary structure PREDiction, PSIPRED Protein Analysis Workbench [1]. FFPred 3 [2] feature of PSIPRED along with PredictProtein [3] and InterPro [4] were used to perform predictions about protein function. Transmembrane domains were predicted using the TMHMM 2.0 server [5] and MEMSTAT [6] module on PSIPRED. Signal peptide was detected using SignalP 5.0 [7] and Phobius [8] and the hydrophobicity indices were calculated using ProtScale [8]. DISULFIND [9] and DiANNA [10] were used to predict disulfide bonds. Glycosylation site predictions were performed using GlycoMine [11], GlycoEP [12], and NetOGlyc [13,14]. CLUSTALW [15] was used to perform multiple sequence alignments applying default parameters on the webserver and alignments were visualized using BOXSHADE3.21 [16] and ESPript [17].

1. Buchan, D.W.A.; Jones, D.T. The PSIPRED Protein Analysis Workbench: 20 years on. *Nucleic Acids Res.* **2019**, *47*, W402–W407, doi:10.1093/nar/gkz297.
2. Cozzetto, D.; Minneci, F.; Currant, H.; Jones, D.T. FFPred 3: Feature-based function prediction for all Gene Ontology domains. *Sci. Rep.* **2016**, *6*, 1–11, doi:10.1038/srep31865.
3. Yachdav, G.; Kloppmann, E.; Kajan, L.; Hecht, M.; Goldberg, T.; Hamp, T.; Hönigschmid, P.; Schafferhans, A.; Roos, M.; Bernhofer, M.; et al. PredictProtein - An open resource for online prediction of protein structural and functional features. *Nucleic Acids Res.* **2014**, *42*, 337–343, doi:10.1093/nar/gku366.
4. Hunter, S.; Apweiler, R.; Attwood, T.K.; Bairoch, A.; Bateman, A.; Binns, D.; Bork, P.; Das, U.; Daugherty, L.; Duquenne, L.; et al. InterPro : the integrative protein signature database. *Nucleic Acids Res.* **2009**, *37*, 211–215, doi:10.1093/nar/gkn785.
5. Krogh, A.; Larsson, È.; Heijne, G. Von; Sonnhammer, E.L.L. Predicting Transmembrane Protein Topology with a Hidden Markov Model : Application to Complete Genomes. *J. Mol. Biol.* **2001**, *305*, 567–580, doi:10.1006/jmbi.2000.4315.
6. Nugent, T.; Jones, D.T. Transmembrane protein topology prediction using support vector machines. *BMC Bioinformatics* **2009**, *10*, 1–11, doi:10.1186/1471-2105-10-159.
7. Juan, J.; Armenteros, A.; Tsirigos, K.D.; Sønderby, C.K.; Petersen, T.N.; Winther, O.; Brunak, S.; Heijne, G. Von; Nielsen, H. SignalP 5 . 0 improves signal peptide predictions using deep neural networks. *Nat. Biotechnol.* **2019**, *37*, 420–423, doi:10.1038/s41587-019-0036-z.
8. Gasteiger, E.; Hoogland, C.; Gattiker, A.; Duvaud, S.; Wilkins, M.R.; Appel, R.D.; Bairoch, A. Protein Identification and Analysis Tools on the ExPASy Server. *Proteomics Protoc. Handb.* **2005**, 571–607.
9. Ceroni, A.; Passerini, A.; Vullo, A.; Frasconi, P. DISULFIND : a disulfide bonding state and cysteine connectivity prediction server. *Nucleic Acids Res.* **2006**, *34*, 177–181, doi:10.1093/nar/gkl266.
10. P.Clote, F.F. DiANNA 1 . 1 : an extension of the DiANNA web server for ternary cysteine classification. *Nucleic Acids Res.* **2006**, *34*, 182–185, doi:10.1093/nar/gkl189.
11. Li, F.; Li, C.; Wang, M.; Webb, G.I.; Zhang, Y.; Whisstock, J.C.; Song, J. GlycoMine : a machine learning-based approach for predicting N- , C- and O-linked glycosylation in the human proteome. *Bioinformatics* **2015**, *31*, 1411–1419, doi:10.1093/bioinformatics/btu852.
12. Chauhan, J.S.; Rao, A.; Raghava, G.P.S. In silico Platform for Prediction of N- , O- and C-Glycosites in Eukaryotic Protein Sequences. *PLoS One* **2013**, *8*, 1–10, doi:10.1371/journal.pone.0067008.
13. Steentoft, C.; Vakhrushev, Y.; Joshi, H.J.; Kong, Y.; Vester-christensen, M.B.; Schjoldager, K.T.; Lavrsen, K.; Dabelsteen, S.; Pedersen, N.B.; Marcos-silva, L.; et al. Precision mapping of the human O -GalNAc glycoproteome through SimpleCell technology. *EMBO J.* **2013**, *32*, 1478–1488, doi:10.1038/emboj.2013.79.
14. R. Gupta, E.J. and S.B. Prediction of N-glycosylation sites in human proteins Available online: http://www.cbs.dtu.dk/services/NetNGlyc/.

15. Thompson, J.D.; Higgins, D.G.; Gibson, T.J. CLUSTAL W: Improving the sensitivity of progressive multiple sequence alignment through sequence weighting, position-specific gap penalties and weight matrix choice. *Nucleic Acids Res.* **1994**, *22*, 4673–4680, doi:10.1093/nar/22.22.4673.

16. Albà, M. Making alignments prettier. *Genome Biol.* **2000**, *1*, reports2047, doi:10.1186/gb-2000-1-2-reports2047.

17. Robert, X.; Gouet, P. Deciphering key features in protein structures with the new ENDscript server. *Nucleic Acids Res.* **2014**, *42*, 320–324, doi:10.1093/nar/gku316.
